# Appraisal on the Wound Healing Potential of *Deverra tortuosa* DC. and *Deverra triradiata* Hochst Essential Oil Nanoemulsion Topical Preparation

**DOI:** 10.3389/fphar.2022.940988

**Published:** 2022-07-26

**Authors:** Reem A. Kamel, Mohammed S Teiama, Ali M. El-Hagrassi, Sabah H. Elgayed, Mohamed A. Khattab, Elsayed K. El-Sayed, Magda T. Ibrahim, Mohamed S. Mady, Fatma A. Moharram

**Affiliations:** ^1^ Mansheyat El-Bakry, General Hospital, Heliopolis, Egypt; ^2^ Department of Pharmaceutics and Industrial Pharmacy, Faculty of Pharmacy, Helwan University, Cairo, Egypt; ^3^ Department of Phytochemistry and Plant Systematics, Pharmaceutical Industries Division, National Research Centre, Giza, Egypt; ^4^ Department of Pharmacognosy, Faculty of Pharmacy, 6^th^October University, Cairo, Egypt; ^5^ Department of Pharmacognosy, Faculty of Pharmacy, Cairo University, Cairo, Egypt; ^6^ Department of Cytology and Histology, Faculty of Veterinary Medicine, Cairo University, Giza, Egypt; ^7^ Department of Pharmacology and Toxicology, Faculty of Pharmacy, Helwan University, Cairo, Egypt; ^8^ Department of Pharmacognosy, Faculty of Pharmacy, Al -Azhar University, Cairo, Egypt; ^9^ Department of Pharmacognosy, Faculty of Pharmacy, Helwan University, Cairo, Egypt

**Keywords:** *Deverra* species, hydrodistillation, microwave-assisted hydrodistillation, supercritical fluid extraction, nanoemulsion, wound healing

## Abstract

*Deverra tortuosa* (Desf.) DC. and *Deverra. triradiata* Hochst. ex Bioss are perennial desert shrubs widely used traditionally for many purposes and they are characteristic for their essential oil. The objective of the present study was to investigate the *in vivo* wound healing activity of the essential oil (EO) of *D. tortuosa* and *D. triradiata* through their encapsulation into nanoemulsion. EO nanoemulsion was prepared using an aqueous phase titration method, and nanoemulsion zones were identified through the construction of phase diagrams. The EO was prepared by hydrodistillation (HD), microwave-assisted hydrodistillation (MAHD), and supercritical fluid extraction (SFE) and analyzed using GC/MS. *D. tortuosa* oil is rich in the non-oxygenated compound, representing 74.54, 73.02, and 41.19% in HD, MADH, and SFE, respectively, and sabinene represents the major monoterpene hydrocarbons. Moreover, *D. triradiata* is rich in oxygenated compounds being 69.77, 52.87, and 61.69% in HD, MADH, and SFE, respectively, with elemicin and myristicin as major phenylpropanoids. Topical application of the nanoemulsion of *D. tortuosa* and *D. triradiata* (1% or 2%) exhibited nearly 100% wound contraction and complete healing at day 16. Moreover, they exhibit significant antioxidant and anti-inflammatory effects and a significant increase in growth factors and hydroxyproline levels. Histopathological examination exhibited complete re-epithelialization accompanied by activated hair follicles and abundant collagen fibers, especially at a concentration of 2%. Therefore, the incorporation of the two *Deverra* species into nanoemulsion could professionally endorse different stages of wound healing.

## 1 Introduction

Wound formation is a result of interruptions or defects in the skin or mucous membranes of epidermis due to physical or thermal damages (Singh et al., 2013). Wounds are categorized into acute or chronic ones. The acute ones are recovered in a short period, and the wound depth, size, and injury degree represent the factors affecting the healing process. Nonetheless, the chronic wound-healing process takes a long time and is different from that of acute wounds (Schreml et al., 2010). Acute wound healing takes place in a normal, orderly, and timely manner throughout the entire process. Conversely, chronic trauma repair in this manner is considered a challenge, and it is difficult to restore normal anatomical structure and function (Tarnuzzer and Schultz, 1996). In reaction to this damage, the organism initiates the complex wound-healing process that involves, cellular, molecular, and physiological mechanisms, which play a role in either partial or complete tissue repair (Agha et al., 2011). This process is categorized into three interdependent and overlying phases, and each one has its period and particular tissues and cell lines (Gurtner et al., 2008; Sorg et al., 2017; Wang et al., 2018).

The first one is the exudative or inflammatory phase through which a clot is formed to stop the hemorrhage, followed by vasodilation and immune defense mechanism activation (Martin and Leibovich, 2005; Enoch and Leaper, 2008). The proliferative or reconstruction phase of the epidermal, endothelial, and then fibroblasts (Wilgus, 2008) creates initial granulation tissue (Diegelmann and Evans, 2004) and angiogenesis occurs (Gurtner et al., 2008). The last phase is the remodeling phase through which the granular tissue is remodeled through the generation of new collagen fibers and differentiation of fibroblasts takes place in myofibroblasts, resulting in tensile strength increase and allowing the approximation of lesion end (Diegelmann and Evans, 2004; Gurtner et al., 2008). As a result of the wound-healing process complexity, the selection of a suitable wound dressing is essential and the best wound dressing should improve the healing, giving the least benefit to the patient (Martin and Leibovich, 2005; Enoch and Leaper, 2008), and allow faster healing without being too expensive (Diegelmann and Evans 2004; Wilgus, 2008). Also, traditional methods for the treatment of wounds cannot heal 70% of the patients (Thakur et al., 2007). Therefore, to overcome these disadvantages, active wound dressing has been established by incorporating active agents in wound dressing materials to prevent microorganisms from infecting the wound (Martin and Leibovich, 2005). Recently, nanoparticles prepared from silver, gold, and zinc have been widely used in wound-healing preparation. Different nanomaterials can be used for wound healing, among which nanoemulsions possess numerous advantages above other colloidal drug carriers due to their ease and low cost of preparation, as well as, their nanosized droplet diameters and physical/thermodynamic stability (Allam et al., 2018). Moreover, nanoemulsions are considered stable, thermodynamically lipid-based drug delivery systems that consist of oil, surfactant, co-surfactant, and water with a droplet size with a maximum diameter of 100 nm (Shukla et al., 2018). Nanomaterials for wound healing mainly work in one of two common ways. The first way represents the characteristic power of nanomaterials as it helps in the wound closure mechanism. The second way shows its ability to act as a carrier for different therapeutic agents (Hamdan et al., 2017).

Medicinal plants as well as extracts and metabolites isolated from them have enormous potential for wound treatment and management, and a large number of them are used in many countries for tribal and folklore to treat wounds (Thakur et al., 2007). The importance of the essential oil (EO) prepared from different plant sources has made them highly valued in the food and cosmetic industries and in pharmaceutical applications. Moreover, its potent antioxidants, metal chelators, and anti-inflammatory effects that were reported in the preclinical studies gave its possible use in new drug development (Miguel 2010; Silva et al., 2013; Orchard, and van Vuuren 2017). Therefore, based on their biological activities, the essential oil of *D*. *Tortuosa* and *D. triradiata* could be a potential source for wound-healing preparation.

Genus *Deverra* DC. (Syn *Pituranthosus* Viv) belonging to the family Apiaceae comprises about only 13 taxa: nine species and four subspecies among which *D*. *tortuosa* (Desf.) DC. and *D. triradiata* Hochst. ex Bioss which are widely grown in South Sinai, Egypt (Täckholm, 1974). Genus *Deverra* is used traditionally by Egyptians as carminative drinks and for improving stomach pain as well as antiasthmatic and for intestinal parasites (El Mesallamy et al., 2021). *D. tortuosa* (Desf.) DC (Syn. *Pituranthos tortuosus* (Desf.) Benth. and Hook. f. ex Asch. and Schweinf) is a perennial desert fragrant shrub, it grows widely in sandy areas in Arabian share-ecoregion including Egypt (Boulos 2000). It has several uses in traditional medicine as analgesic, carminative, antiasthmatic, diuretic, as well as, it was used in case of stomach pain, intestinal parasites, rheumatism, fever, diabetes, hepatitis, and hypertension (Mahran et al., 1989; Vérité et al., 2004; Krifa et al., 2016; Elshibani et al., 2020; Guetat, 2022) and menstrual regulation (Ashkenazy et al., 1983). Moreover, *D. tortuosa* is used as an edible food due to its medicinal and aromatic value. Essential oils of *D. tortuosa* prepared by hydrodistillation was mentioned before (Mahran et al., 1989; Abdel-Ghani and Hafez 1995; Singab et al., 1998; Abdallah and Ezzat, 2011; Mostafa et al., 2020; Fayed et al., 2021). Moreover, the previous reports investigate antimicrobial, antioxidant, allelopathic (Fayed et al., 2021), anticancer (Abdallah and Ezzat, 2011), and antibacterial (Singab, 2003). *D. triradiata* Hochst. ex Boiss (Syn. *Pituranthos triradiatus*) is a glabrous desert leafless shrub widely grown in the Mediterranean coastal region and the south Sinai Peninsula. It is traditionally used by Bedoons in cases of stomach pains, intestinal parasites, bloody cough, haematuria, and for menstruation regulation (Halim et al., 1991). There is little information about the analysis and biological activity of *D. triradiata* except the evaluation of its anti-inflammatory activity (Donia et al., 2015). Taking into consideration the biological activities of the EO of *D. tortuous* and *D. triradiata,* they could be a potential applicant to make the wound-healing process more efficient. So our objectives are i) preparation of the EO of two *Deverra* species using a different method; ii) preparation of nanoemulsion from the two oils; and iii) evaluation of the wound healing ability of the two nanoemulsion oils.

## 2 Material and Methods

### 2.1 Plants Material

Aerial parts of *D*. *tortuosa* (Desf.) DC and *D. triradiata* Hochst. ex Bioss were collected during February 2021 from Wadi Degla protectorate, Cairo, Egypt. The two species were authenticated by Prof. Dr. Abduo Marie Hamed, professor of Plant Ecology, Faculty of Science, Al-Azhar University, Nasr city. A voucher specimen (01 DTO 2021 and 02 DTI 2021) is kept at the Departement of Pharmacognosy, Faculty of Pharmacy, Al-Azhar University, Egypt.

### 2.2 Chemical and Reagents

Oleic acid and polysorbate 80, were purchased from Al-Nasr for chemicals (Abou-Zabal, Cairo, Egypt) and propanol alcohol was supplied from Algoumhoria Company for chemicals (Garden City, Cairo, Egypt). Jojoba oil was purchased from Chemajet Company (New Borg El-Arab City, Borg Al Arab, Alexandria, Egypt). Mebo^®^ ointment is a well-known marketed product for wound healing (Julfar, UAE, active ingredients: *β*-sitosterol, berberine, and baicalin in sesame oil and beeswax base).

### 2.3 Essential Oils Isolation

#### 2.3.1 Hydrodistillation (HD) Method

Fresh aerial parts of *D*. *tortuosa* and *D. triradiata* (250 g, each) were placed in a round-bottom flask and mixed with distilled, deionized water, then subjected to hydrodistillation by Clevenger apparatus for 5 h to isolate their essential oils. (Denys and Simon, 1990).

#### 2.3.2 Microwave-Assisted Hydrodistillation (MAHD)

MAHD was performed using CEM Corporation, Matthews, NC, United States, model (MARS 240/50, No. 907511) microwave oven. It is a multimode microwave (2,450 MHz) with a maximum delivered power of 1200 W. About 250 g of both species were placed in a 1 L flask together with 500 ml of deionized water and then put within the microwave oven cavity. This mixture was heated at a fixed power of 800 W at 100 °C for 60 min to collect the released oil (Ghazanfar et al., 2020).

#### 2.3.3 Supercritical Fluid Extraction (SFE)

Supercritical CO_2_ gas was used for oil extraction (Spe-ed TM SFE-2/4, applied separations, built in conjunction with the USDA1, United States). Dry aerial parts of two species (200.0 g, each) were extracted with SFE under 150 bar pressure and 40 °C temperature for 60 min in static and dynamic condition, respectively, for 3 h. The oil prepared from the three methods was dried using anhydrous Na_2_SO_4_ and kept in a refrigerator till analysis (Suetsugu et al., 2013).

The percentage of oil content was calculated as essential oil volume (ml) per 100 g of fresh plant material.

### 2.4 GC/MS Analysis of Essential Oils

Essential oils analysis was done using gas chromatography (Shimadzu QP2010) coupled with a quadrupole mass spectrometer (Shimadzu Corporation, Kyoto, Japan). EO components were separated using Rtx-5MS fused bonded column (Restek, United States, 30 m × 0.25 mm inner diameter x 0.25 μm film thickness) together with a flame ionization detector. The condition of gas chromatography is: Injector temperature is 250°C; initial oven temperature is 45°C for 2 min, then planned to 300°C at a rate of 5°C/min, and then reserved constant at 300°C for 5 min. The detector temperature is 280°C. Helium is used as carrier gas with a 1.41 ml/min flow rate. The samples (1 μL) were injected using split mode (1:15). The MS data was operated as follows: Ion source and interface temperatures are 200 and 280°C respectively, electron ionization mode is 70 eV and the scanning range is 35–500 amu.

Identification of the essential oils components was done by comparing retention indices (RI) of them relative to standard *n*-alkanes (C_8_-C_28_); and their MS to NIST and WILEY mass spectral library database; (similarity index >90%) (Adams 2005; Gad et al., 2021).

### 2.5 Construction of Pseudo-ternary Phase Diagram for *D*. *tortuosa* and *D. triradiata* Essential Oils

It was initiated to prepare various nanoemulsion formulations with different concentrations from the two *Deverra* species. The diagram was developed using the aqueous phase titration method (Shakeel, et al., 2008; Chaudhari and Kuchekar, 2018). Briefly, oleic acid and jojoba oil mixture as oily phase were mixed with ratio of 1:1 w/w while polysorbate 80 and propanol mixture with different ratio (0.5:3.5, 1:3, 1.5:2.5, 2:2, 2.5:1.5, 3:1, 3.5:0.5, 0.5:4.5, 1:4, 1.5:3.5, 2:3, 2.5:2.5, 3; 2, 3.5:1.5, 4:1, and 4.5:0.5 w/w) was utilized as surfactant/cosurfactant component. The oily phase and surfactant/cosurfactant in ratios of 1:4, 1.5:2.5, 2:3, and 2.5:2.5 were mixed in a glass beaker on a hot plate stirrer then they were titrated with deionized water till resistant turbidity was obtained. All weights were recorded and calculated as weight percentage (w/w %), and the pseudo ternary phase diagram was constructed using Chemix school ternary diagram software.

### 2.6 Preparation of Nanoemulsion for the *D*. *tortuosa* and *D. triradiata* Essential Oils

From the previously constructed pseudo-ternary phase diagram, a certain point of appropriate concentrations of oil (oleic/jojoba mixture), SAA/Co-SAA mixture (tween 80/propanol), and water was selected to prepare the following nanoemulsions. Five different formulae of nanoemulsion were prepared with a spontaneous method of emulsification (Aswathanarayan and Vittal, 2019) with slight modifications. The blank formula (F1) was prepared by mixing oleic acid, jojoba oil, tween 80, and propanol in a glass beaker with a magnet on a hot plate stirrer (Wisestir MSH-20D, Belgium). Then water was added dropwise and kept overnight at room temperature with stirring at 600 rpm. The other four formulae (F2- F5) were prepared with the same procedures in presence of *D. tortuosa* and *D. triradiata* essential oils by concentrations of 1% and 2% w/w from the total weight of emulsion for both (Supplementary Table S1 **)**

### 2.7 Physicochemical Evaluation of Two *Deverra* Species Nanoemulsions

#### 2.7.1 pH Determination

pH values for the plain nanoemulsion and that loaded with two *Deverra* oils were measured using a pH meter (Jenco Large pH/mV/Temperature Meter Kit - 6173KB, United States). The measuring was carried out in triplicate.

#### 2.7.2 Droplet Size Determination and Zeta Potential

The average diameter of nanoemulsion droplets, droplet size distribution, and surface zeta potential were measured for both plain formulae and two *Deverra* species nanoemulsions using the dynamic light scattering (DLS) technique. From each formula, 100 µL was diluted with deionized water and evaluated for droplet size using the light scattering technique of angle 160° at 25 °C (Beckman Coulter Delsa nanoparticle size analyzer, United States). The surface charge was evaluated using clear disposable zeta cells with the same device and results were recorded in triplicate.

### 2.8 *In Vivo* Wound Healing Evaluation

#### 2.8.1 Experimental Animals

Sprague Dawley adult female rats (180–200 g) were supplied from the Egyptian Organization of Biological products and Vaccines breeding unit (Helwan, Egypt). Rats were randomized and kept under controlled environmental conditions at a constant temperature (23°C ± 2°C), maintained in a 12/12-h light-dark cycle, and supplied with free access to a standard pellet diet (Meladco Feed Company, October City, Cairo, Egypt) for 1-week acclimatization before the experimental work and given tap water *ad libitum*. All experimental procedures were approved by the Institutional Animal Ethics Committee guidelines for animal care and use at Al-Azhar University (approval no, AZU: 333-2022), and conducted according to the European Community Directive (86/609/EEC), a national rule on animal care that is consistent with the NIH Guidelines for the Care and Use of Laboratory Animals (8th edition).

#### 2.8.2 Excision Wound Model

Anesthesia of rats was done using ketamine hemisulfate (100 mg/kg, i.p.) (Labib et al., 2019) and their back hair was shaved by an electric shaver and cleaned with 70% ethanol. On the dorsal interscapular region of each rat, a 225 mm^2^ (1.5 cm × 1.5 cm) full-thickness cutaneous square wound was made by removing a skin patch, and wounds were kept undressed until the end of the experiment (Davoodi-Roodbordeii et al., 2019). Lidocaine hydrochloride (2%) containing 1: 80,000 epinephrine (4.4 mg/kg) was *S.C* injected into rats to reduce pain near the wound area immediately after wounding (Koshak et al., 2021). To maintain aseptic conditions, bench surfaces and cages to be used were cleaned by wiping over with a wet cloth that had been soaked with a cleaning agent, followed by disinfection with 70% alcohol. Moreover, instruments used in surgery (forceps, scissors, and scalpel) were held in a dish containing 70% ethanol and were rinsed in normal saline, to remove the ethanol before re-introduction into rat tissues.

#### 2.8.3 Experimental Design

Thirty-six wounded rats (1rat/cage) were randomly divided into six groups (n = 6) as follows.

Group I is served as wound control; group II and III: *D. tortuosa* nanoemulsion 1 and 2%; group IV and V: *D. triradiata* nanoemulsion 1 and 2%; group VI: Standard control (Mebo^®^ ointment).

The nanoemulsion of *D. tortuosa*, *D. triradiata* oil, and Mebo^®^ was topically applied once daily for 16 days on the wounded areas of the respective groups, while the plane base was applied for the wounded control group. The rats were observed daily for well-being and those that showed any sign of infection were excluded from the study (Note: in the current study, no infected wounds were observed and the mortality rate was equal to zero).

#### 2.8.4 Determination of the Surface Area

The wound surface area was calculated by tracing the wound margin on a transparent sheet using a permanent marker (Kundu et al., 2016). After that, the tracing sheet was placed on graph paper (1-mm^2^) and traced out. The wound surface area was calculated every 4 days (4th, 8th, 12th, 16th day). The wound contraction was expressed as a decline of the initial wound area percentage using the following formula.

$$\text{The percentage of wound contraction }=\frac{\text{Inital wound area}-\text{specific day wound area}}{\text{ initial wound area}}\text{ x }100$$

At the end of the experiment (day 17), the rats were sacrificed and the samples of granulation tissue were dissected from the wound site and divided into equal parts. For histopathological analysis, one part of the skin was preserved in neutral formalin (10%) while the remaining parts were kept at -80°C for further biochemical analyses.

#### 2.8.5 Biochemical Analyses

##### 2.8.5.1 Preparation of Skin Tissue Homogenate

Samples of the skin were cautiously rinsed in ice-cold normal saline, then dried with filter papers, and weighed. Ten percent of the homogenate (w/v) was prepared in ice-cold phosphate saline buffer (0.1M, pH 7.4) and then centrifuged for 30 min at 3,000 rpm and 4°C. The supernatant produced was used for the estimation of the biochemical parameters.

##### 2.8.5.2 Determination of Lipid Peroxidation and Antioxidant Markers

The malondialdehyde (MDA) level as a lipid peroxidation marker in the granulation tissue was assessed following the kit’s instructions (Biodiagnostic, Cat. No. MD 25 29, Cairo, Egypt), while the reduced glutathione (GSH) and catalase (CAT) levels as antioxidant markers were evaluated according to the kit’s instructions (Biodiagnostic, Cat. No. GR 25 11 and CA 25 17, Cairo, Egypt, respectively).

##### 2.8.5.3 Determination of Inflammatory Markers

The tumor necrosis factor α (TNF-α) level and interleukin-1β (IL-1β) were assessed using enzyme-linked immunosorbent assay kits ELISA (CUSABIO Life Sciences, Cat. No. CSB-E11987r, Wuhan, China) and (MyBioSource, San Diego, Cat. No. MBS825017, United States) respectively according to the manufacturer’s instructions. Samples were added to appropriate microtiter plate wells coated with monoclonal antibodies (capture antibodies), and any rat TNF-α or IL-1β would bind to the immobilized antibodies. The wells were washed and biotin-conjugated anti-rat TNF-α or IL-1β antibodies (1:100) were added. After a second wash, avidin-horseradish peroxidase (avidin-HRP) was added, producing an antibody-antigen-antibody sandwich. The wells were washed and tetramethylbenzidine (TMB) substrate solution was added, which produced a blue color that was directly proportional to the amount of TNF- α or IL-1β present in the sample. The reaction was terminated by the addition of sulfuric acid, which resulted in a color change from blue to yellow, which was measured spectrophotometrically at 450 nm.

##### 2.8.5.4 Evaluation of Growth Factors

Enzyme-linked immunosorbent assay kits (ELISA) were used to evaluate the levels of transforming growth factor-beta (TGF-β) and vascular endothelial growth factor (VEGF) from My Biosource, Inc. (Cat. No. MBS824788 and MBS724516), respectively. The methods were performed as mentioned under 2.8.5.3.

##### 2.8.5.5 Determination of Hydroxyproline Content

To confirm the results of the wound contraction, the content of hydroxyproline (a major component of the protein collagen) was estimated using ELISA Kits (MyBioSource, Cat. No. MBS017427, San Diego, United States) as stated by the manufacturer’s instructions. The methods were performed as mentioned under 2.8.5.3.

#### 2.8.6 Histopathological Examination

Samples of skin wound tissue was fixed for 48 h in 10% neutral buffered formalin and then dehydrated in serial ascending grades of ethanol, cleared in xylene then infiltrated by synthetic paraplast tissue embedding medium. Tissue sections were made at the middle zones of different wound samples to demonstrate the different skin layers using a rotatory microtome. After that, they were fixed into glass slides and stained with hematoxylin and eosin as microscopic examination staining standards. Moreover, Masson’s trichrome stain is used for quantitative analysis of collagen fibers contents. All standard procedures for sample fixation and staining process were done according to Culling, 2013.

#### 2.8.7 Microscopical Analysis

According to Abbas et al. (2021), six random non-overlapping microscopic fields from each sample per group were scanned and then analyzed for obtaining the mean area percentage of segmented dermal collagen fibers contents in Masson’s trichrome stained section. All data and micrographs were obtained by using a Full HD microscopic imaging system operated by using the Leica application module for histological analysis.

#### 2.8.8 Statistical Analysis

The results were expressed as mean value ±SEM. Statistical analysis and graphical representations were performed using GraphPad Prism, version 8 (GraphPad Software Inc., San Diego, California, United States), by one-way analysis of variance, followed by a Tukey’s test to measure the statistical significance between various groups. The value *p* < 0.001 was considered as significant.

## 3 Result and Discussion

It was reported that the selection of the suitable method for essential oils preparation is a tedious process and depends on several issues (Rassem et al., 2016). The hydrodistillation method is considered the most common and low-priced one, but it is well known that it may affect the oil composition through saponification, isomerization, and or polymerization of the more labile compounds (Koedam et al., 1979). Meanwhile, MADH and SFE are considered superior green technology or climate-friendly producing high-quality essential oil in a short time with minor environmental degradation (Mishra and Rathore 2021). Moreover, SFE produces a higher yield, diffusion coefficient, and lower viscosity oil (Meyer-Warnod, 1984). In the present study, we prepared *D*. *tortuosa* and *D. triradiata* oil using three different techniques *viz* HD and MAHD for the fresh aerial parts and SFE for a dry sample, to highlight the effect of the preparation method on the yield and chemical composition of the oil. From our results, it was found that the method of preparation affects not only the yield of oil being 0.52, 0.2, and 1.75 v/w (*D. tortuosa*) and 0.28, 0.28, and 1.75 v/w (*D. triradiata* aerial part) for HD, MAHD, and SFE respectively, but also the color of the oil is a pale yellow, light yellow, and yellowish-orange respectively in the two *Deverra* species. As shown from the result, it was found that the percentage of oil prepared by SFE represents the highest percentage of HD and MAHD. SFE is a liquid-gas intermediate phase, which owns both liquid and gas properties, low or no surface tension, and a non-viscous (10–100 times less than liquids) nature which allowed high penetration rate and excellent solvation power encouraging faster extraction and worth yield (Sargenti and Lancas, 1997).

### 3.1 GC/MS Analysis for *D. tortuosa* and *D. triradiata* Essential Oils

The influence of essential oil extraction techniques was observed on the qualitative and quantitative composition of *D. tortuosa* and *D. triradiata* oil which was noticeable. Regarding the essential oil of *D. tortuosa* aerial parts, a total number of 25 components constituting 98.06% and 96.05% were identified in HD and MAHD samples respectively, moreover 20 compounds constituting 90.93% were identified in the SFE sample (Table 1, Supplementary Figure S1–S3). Variability in the type and amount of the identified compounds in the *D. tortuosa* samples was noticeable; in the case of HD essential oil, sabinene (40.28%), terpinen-4-ol (20.87%), β-myrcene (12.38%), α-terpinene (4.87%), and γ-terpinene (7.84%) represent the major components, while the same compounds represent the major ones in case of MAHD but with a different percentage being sabinene (42.08%), terpinen-4-ol (19.88%), β-myrcene (10.16%), α-terpinene (2.83%), γ-terpinene (5.53%). However, in the SFE sample, in addition to sabinene (20.60%) and terpinen-4-ol(19.61%) as in HD and MAHD, there are other major components as germacrene D (10.78%), myristicin (6.84%), elemicin (13.53%) and β-eudesmol (5.61%). It was found that these results are consistent with the major constituents of the previously prepared *D. touousa* oil (Singab, 2003; Abdallah and Ezzat, 2011). Moreover, it is interesting to observe that the different preparation methods used influenced the percentage of the chemical class of the oil. As shown from the result, there is a difference was noticed in the *D. tortuosa* oil, where is the percentage of oxygenated compounds being 23.52, 23.03, and 49.74 for HD, MADH, and SFE, respectively, and the percentage of non-oxygenated compounds was 74.54 (HD), 73.02 (MADH), and 41.19 (SFE). In addition, there is another interesting difference in the chemical class of the compounds was observed in the percentage of monoterpene hydrocarbon (MH) and sesquiterpene hydrocarbons (SH) where the lowest percentage of MH (25, 79%) and the highest one of SH (14.03%) was found in case of SFE sample, also it contains the lowest percentage of the oxygenated sesquiterpene (7.40%), phenylpropanoids (21.09%), and the oxygenated monoterpenes (OM) being 21.25%. As in the case of *D. tortuosa*, the effect of the preparation method was observed in the case of *D. triradiata* aerial parts essential oil; a total number of 30 components representing 95.89 % and 95.52% were identified in HD and MAHD, respectively, while only 18 compounds constituting 97.60% were detected in SFE sample (Table 2, Supplementary Figure S4–S6). In addition, the variability in the type and amount of the detected compounds in the essential oil of *D. triradiata* was obvious; It was found that elemicin, germacrene D, and myristicin represent the major components in the *D. triradiata* sample but their percentage was differing in HD, MAHD, and SEF*.* The percentage of elemicin is 33.43%, myristicin 20.23%, and germacrene D is 10.34 in the HD sample, while in MAHD, germacrene D, elemicin, and myristicin are represented by 24.80%, 21.97%, and 18.23%, respectively, as well as their percentage, is 34.83%, 23.30 %, and 12.55% for elemicin, germacrene D, and myristicin in SFE, respectively. Moreover, the used preparation method affected the chemical class percentage of the oil as mentioned in the result, there is a difference in the percentage of oxygenated compounds being 69.77, 52.87, and 61.69 for HD, MADH, and SFE, respectively, moreover, the non-oxygenated compounds represent 26.12, 42.65, and 35.91%in HD, MADH, and SFE, respectively. In addition, there is a difference in the percentage of monoterpene hydrocarbons (MH) and sesquiterpene hydrocarbons (SH) where SH represents the largest percentage in all the three methods being 14.03, 32.51, and 29.16% in HD, MADH, and SFE, respectively, moreover, the aromatic hydrocarbons are representing in the highest percentage in case of HD (8.22%). Regarding the oxygenated compounds, it was found that phenylpropanoid represents a large percentage in the three methods HD (56.04%), MADH (41.83%), and SFE (47.38%). The structure of the compounds identified in *D*. *tortuosa* and *D. triradiata* oil are represented in Supplementary Figure S7, S8, respectively. It was found that there is a variation in the amount and number of the compounds identified in the essential oil of both Deverra species and other previously reported species which could be attributed to the environmental condition and genetic variation**.** Furthermore, the drying period, harvesting time, and temperature affected the EO both qualitatively and quantitatively**.** Moreover, since Deverrais is a perennial plant so other factor in plant age may play a significant role in the chemical composition of the EO (Carvalho Filho et al., 2006; Patel et al., 2016).

**TABLE 1:** Identified chemical composition of *D. tortuosa* aerial parts essential oil extracted by different methods of extraction (HD, MAHD, SFE).

Peak	R_t_	Compound	M.F	RI_exp_	RI_lit_	Content %			Identifications
						HD	MAHD	SFE	
1	7.10	*α*-Thujene	C_10_H_16_	907	907	0.87	0.52	-----	MS,RI
2	7.29	*α*-Pinene	C_10_H_16_	914	914	0.23	0.16	-----	MS,RI
3	7.72	Camphene	C_10_H_16_	930	930	0.05	-----	-----	MS,RI
4	8.59	Sabinen	C_10_H_16_	961	961	40.28	42.08	20.60	MS,RI
5	8.64	*β*-Pinene	C_10_H_16_	963	963	0.57	0.81	-----	MS,RI
6	9.07	*β*-Myrcene	C_10_H_16_	979	979	12.38	10.16	2.46	MS,RI
7	9.43	*α*-Phellandrene	C_10_H_16_	992	993	0.37	0.20	-----	MS,RI
8	9.60	3-Carene	C_10_H_16_	998	998	0.09	0.06	-----	MS,RI
9	9.81	*α*-Terpinene	C_10_H_16_	1,005	1,005	4.87	2.80	0.49	MS,RI
10	10.06	*p*-Cymene	C_10_H_14_	1,013	1,013	1.07	4.83	1.37	MS,RI
11	10.18	Sylvestrene	C_10_H_16_	1,017	1,019	2.40	1.52	0.73	MS,RI
12	10.48	*trans*-*β*-Ocimene	C_10_H_16_	1,027	1,027	1.36	1.07	-----	MS,RI
13	11.15	*γ*-Terpinene	C_10_H_16_	1,048	1,048	7.84	5.53	1.51	MS,RI
14	12.04	*p-Mentha-2,4(8)-diene*	C_10_H_16_	1,077	1,077	1.64	1.07	-----	MS,RI
15	12.93	*α*-Thujone	C_10_H_16_O	1,105	1,105	0.03	0.07	-----	MS,RI
16	13.08	*trans*-*p*-2-Menthen-1-ol	C_10_H_18_O	1,110	1,114	1.08	1.22	0.92	MS,RI
17	13.65	*cis*-*p*-Menth-2-en-1-ol	C_10_H_18_O	1,129	1,129	0.75	0.87	0.72	MS,RI
18	14.90	Terpinen-4-ol	C_10_H_18_O	1,169	1,169	20.87	19.88	19.61	MS,RI
19	15.24	*α*-Terpineol	C_10_H_18_O	1,180	1,180	0.55	0.37	------	MS,RI
20	16.82	*p*-Cymene-2-ol methyl ether	C_11_H_16_O	1,233	1,233	0.10	0.31	------	MS,RI
21	18.03	Bornyl acetate	C_12_H_20_O_2_	1,276	1,276	0.10	0.07	------	MS,RI
22	20.58	*α*-Copaene	C_15_H_24_	1,365	1,365	0.03	0.14	0.36	MS,RI
23	20.82	(-)-*β*-Bourbonene	C_15_H_24_	1,373	1,373	------	------	1.31	MS,RI
24	20.99	(-)-*β*-Elemene	C_15_H_24_	1,379	1,379	------	------	1.24	MS,RI
25	23.42	Germacrene D	C_15_H_24_	1,472	1,472	0.42	1.84	10.78	MS,RI
26	23.57	*β*-Selinene	C_15_H_24_	1,478	1,478	0.07	0.23	0.34	MS,RI
27	24.48	Myristicin	C_11_H_12_O_3_	1,513	1,514	0.04	0.07	6.84	MS,RI
28	25.30	Elemicin	C_12_H_16_O_3_	1,545	1,545	------	------	13.53	MS,RI
29	25.89	Spathulenol	C_15_H_24_O	1,568	1,568	------	------	0.47	MS,RI
30	27.03	Dill apiol	C_12_H_14_O_4_	1,614	1,615	------	------	0.72	MS,RI
31	27.68	*β*-Eudesmol	C_15_H_26_O	1,642	1,642	------	0.17	5.61	MS,RI
32	28.49	Alloaromdendrene oxide-(2)	C_15_H_24_O	1,678	1,678	------	------	1.32	MS,RI
Total identified compounds	-	-	-	-	-	98.06	96.05	90.93	-
Non-oxygenated compounds	Monoterpene hydrocarbons (MH)	-	-	-	-	72.95	65.98	25.79	-
	Sesquiterpene hydrocarbons (SH)	-	-	-	-	0.52	2.21	14.03	-
	Aromatic hydrocarbons (ArH)	-	-	-	-	1.07	4.83	1.37	-
Oxygenated compounds	Oxygenated monoterpenes (OM)	-	-	-	-	23.48	22.79	21.25	-
	Oxygenated sesquiterpenes (OS)	-	-	-	-	-------	0.17	7.40	-
	Phenylpropanoid (PP)	-	-	-	-	0.04	0.07	21.09	-

t_R_, retention time; RI_exp._, experimental refractive index; RI_lit_, reference refractive index; MF, molecular formula.

**TABLE 2:** Chemical composition of *D. triradiata* aerial parts oil extracted by HD, MAHD and SFE.

Peak	Rt	Compound	M.F	RIexp	RIlit	Content %			Identifications
						HD	MAHD	SFE	
1	7.29	α-Pinene	C_10_H_16_	914	914	1.05	0.54	-----	MS,RI
2	7.72	Camphene	C_10_H_16_	930	930	0.31	0.15	-----	MS,RI
3	8.48	Sabinen	C_10_H_16_	957	957	0.46	1.38	-----	MS,RI
4	8.56	β-Pinene	C_10_H_16_	960	961	0.14	0.13	-----	MS,RI
5	9.05	β-Myrcene	C_10_H_16_	978	978	------	0.10	-----	MS,RI
6	9.43	α-Phellandrene	C_10_H_16_	992	993	0.03	--------	-----	MS,RI
7	10.08	p-Cymene	C_10_H_14_	1,014	1,014	8.22	6.22	0.90	MS,RI
8	10.19	β-Phellandrene	C_15_H_24_	1,017	1,017	1.74	1.56	-----	MS,RI
9	18.04	Bornyl acetate	C_15_H_24_	1,276	1,267	1.57	0.45	-----	MS,RI
10	20.58	α-Copaene	C_15_H_24_	1,365	1,365	0.19	0.56	0.44	MS,RI
11	20.84	(-)-β-Bourbonene	C_15_H_24_	1,374	1,374	0.95	2.28	2.16	MS,RI
12	21.02	(-)-β-Elemene	C_15_H_24_	1,380	1,380	0.97	2.30	1.63	MS,RI
13	22.05	β-Copaene	C_15_H_24_	1,418	1,421	0.13	0.33	0.31	MS,RI
14	22.45	γ-Muurolene	C_15_H_24_	1,434	1,435	0.08	0.21	0.20	MS,RI
15	22.70	α-Humulene	C_15_H_24_	1,444	1,444	0.05	0.15	-----	MS,RI
16	23.48	Germacrene D	C_15_H_24_	1,474	1,474	10.34	24.80	23.30	MS,RI
17	23.59	β-Selinene	C_15_H_24_	1,478	1,478	0.12	0.22	0.43	MS,RI
18	23.84	β-Guaiene	C_15_H_24_	1,488	1,488	0.58	1.08	0.42	MS,RI
19	24.28	δ- Cadinene	C_15_H_24_	1,505	1,505	0.09	0.11	-----	MS,RI
20	24.61	Myristicin	C_11_H_12_O_3_	1,518	1,518	20.23	18.23	12.55	MS,RI
21	24.74	β-Vatirenene	C_15_H_24_	1,523	1,527	0.14	0.16	------	MS,RI
22	25.17	Germacrene B	C_15_H_24_	1,540	1,540	0.39	0.31	0.27	MS,RI
23	25.47	Elemicin	C_12_H_16_O_3_	1,552	1,552	33.43	21.97	34.83	MS,RI
24	25.95	Spathulenol	C_15_H_24_O	1,570	1,570	0.88	0.76	0.99	MS,RI
25	26.16	β-Copaene-4α-ol	C_15_H_24_O	1,579	1,579	0.13	-----	-----	MS,RI
26	26.33	Salvial-4 (14)-en-1-one	C_15_H_24_O	1,585	1,586	-----	0.36	-----	MS,RI
27	27.05	Dill apiol	C_12_H_14_O_4_	1,615	1,615	1.85	1.32	-----	MS,RI
28	27.23	7-epi-γ-Eudesmol	C_15_H_26_O	1,623	1,622	0.16	0.11	-----	MS,RI
29	27.68	β-Eudesmol	C_15_H_26_O	1,642	1,642	9.21	7.60	8.94	MS,RI
30	28.40	Apiol	C_12_H_14_O_4_	1,674	1,674	0.53	0.31	------	MS,RI
31	28.54	Alloaromadendrene oxide-(2)	C_15_H_24_O	1,680	1,678	1.78	1.76	2.30	MS,RI
32	36.89	Linoleic acid, methyl ester	C_19_H_34_O_2_	2077	2076	------	-----	1.05	MS,RI
33	36.99	Oleic acid, methyl ester	C_19_H_36_O_2_	2083	2085	------	------	1.03	MS,RI
34Total identified compounds	49.87	2-methyloctacosane	C_29_H_60_	2,855	2,857	0.14	0.06	5.85	MS,RI
						95.89	95.52	97.60	
Non-oxygenated compounds	Monoterpene hydrocarbons (MH)	-	-	-	-	3.73	3.86	------	-
	Sesquiterpene hydrocarbons (SH)	-	-	-	-	14.03	32.51	29.16	-
	Aromatic hydrocarbons (ArH)	-	-	-	-	8.22	6.22	0.90	-
	Aliphatic hydrocarbons (AH)	-	-	-	-	0.14	0.06	5.85	-
Oxygenated compounds	Oxygenated monoterpenes (OM)	-	-	-	-	1.57	0.45	------	-
	Oxygenated sesquiterpenes (OS)	-	-	-	-	12.16	10.59	12.23	-
	Phenylpropanoid (PP)	-	-	-	-	56.04	41.83	47.38	-
	Acyclic compounds (AC)	-	-	-	-	-------	------	2.08	-

tR, retention time; RI_exp._, experimental refractive index; RI_lit_, reference refractive index; MF, molecular formula.

### 3.2 Preparation of Nanoemulsion for *D*. *tortuosa* and *D. triradiata* Essential Oils

As it was shown from the essential oil yield prepared by HD, MADH, and SFE, it was found that SFE produces a large percentage (1.75%), so it was used for the nanoemulsion preparation and further wound-healing evaluation.

#### 3.2.1 Construction of Pseudo-ternary Phase Diagram for *D*. *tortuosa* and *D. triradiata* Essential Oils

**FIGURE 1:**
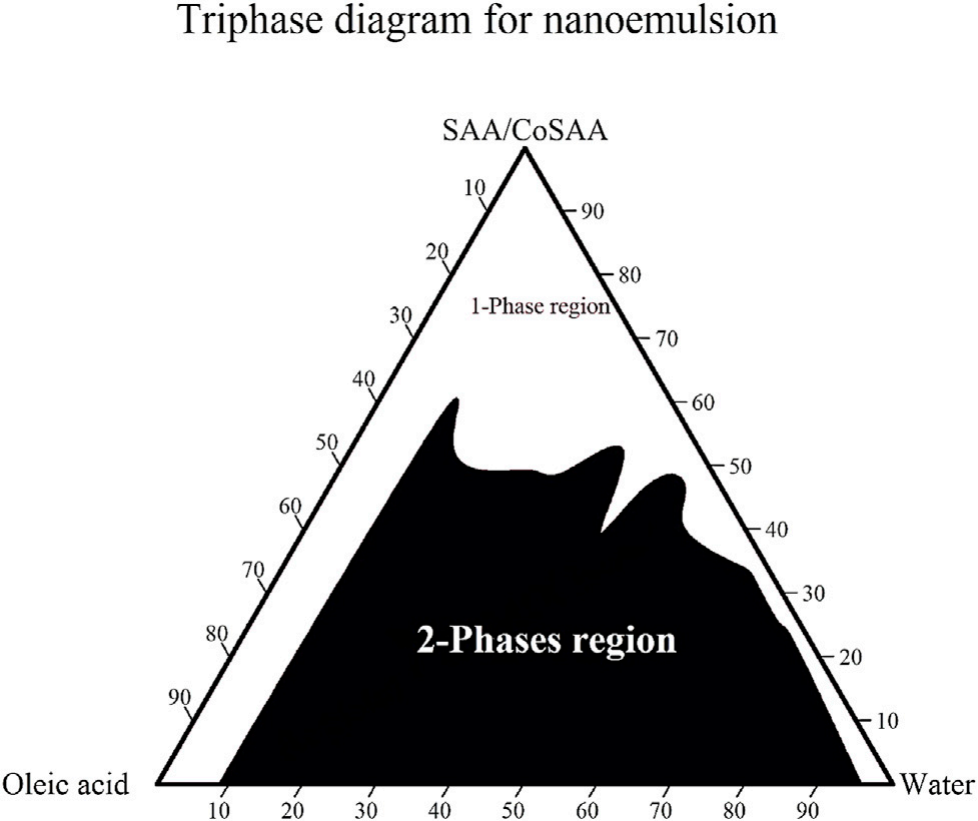
Ternary phase diagram for oleic acid, and water in presence of Tween 80 and isopropyl alcohol as SAA/CoSAA mixture.

Pseudo-ternary phase diagrams were constructed using EEO, oleic/jojoba mixture, tween 80/propanol, and water for the development of EEO nanoemulsions. As a result of the ternary phase diagram (Figure 1), the results summary of aqueous phase titration is represented in Supplementary Table S2. It was found that the white area revealed the ability of the SAA/CoSAA mixture to decrease interfacial tension between water and oleic acid to give one phase clear homogenate while the black area represents the two phases region at which the SAA/CoSAA mixture was not able to break out the tension between the water and oleic acid. Therefore, we select a certain point at the one-phase region and near separating line borders to get a homogenous one-phase nanoemulsion that is used as a base for *D. tortuosa and D. triradiata* essential oils with concentrations of 1%, and 2% for each oil.

#### 3.2.2 pH Determination

**FIGURE 2:**
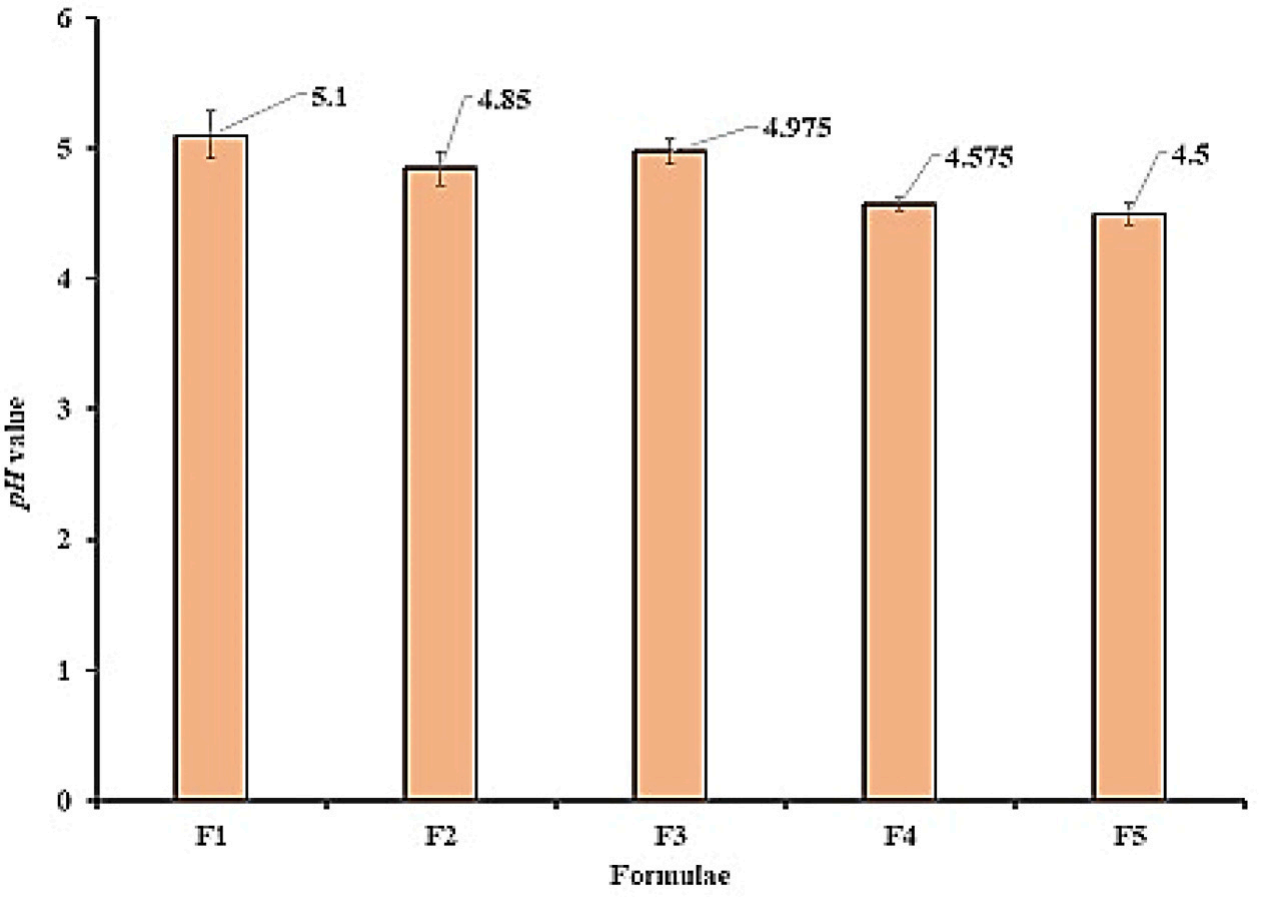
pH values for all prepared formula.

pH value of all samples is in the range of 4.5 ± 0.082 to 5.1 ± 0.183 (Figure 2) which is compatible with that of normal skin (4–6) to avoid skin irritation upon application (Ali and Yosipovitch., 2013).

#### 3.2.3 Droplet Size, Polydispersity Index, and Zeta Potential

**FIGURE 3:**
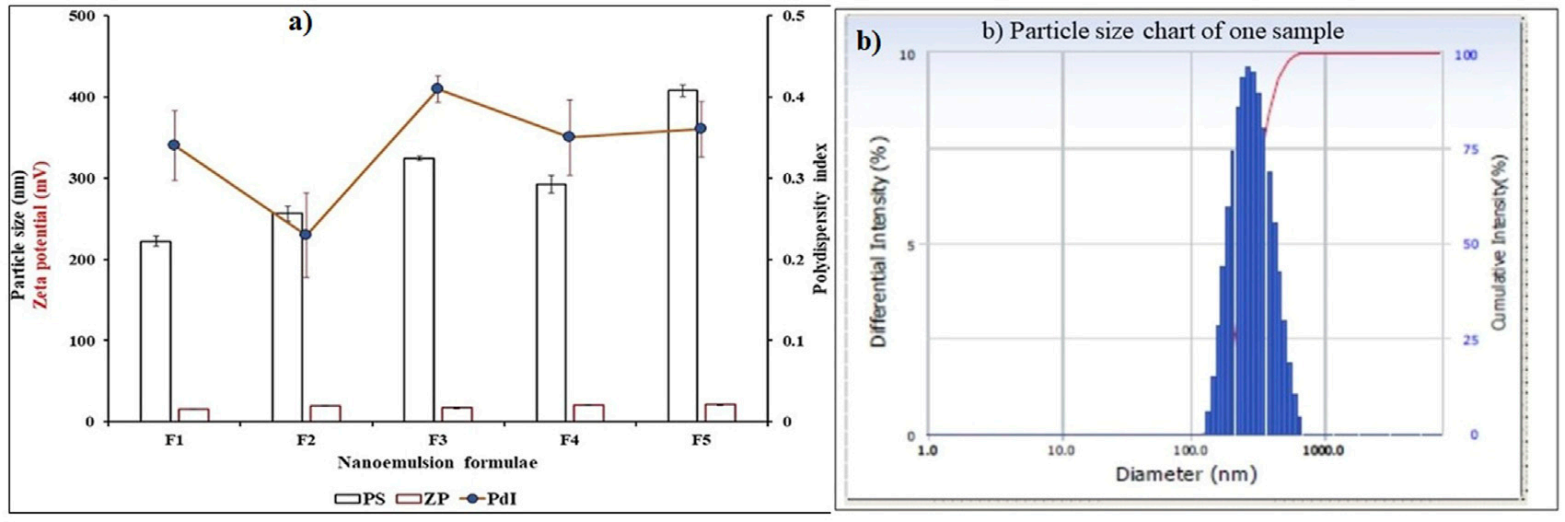
**(A)** Particle size, polydispersity index and Zeta-potential for blank and oil loaded formula, **(B)** Particle size chart of one sample.

The droplet size for blank and oil-loaded formulas ranges from 224.43 ± 5.77 to 407.7 ± 7.27 nm Table 3 and Figure. Moreover, the oil-loaded formulae showed globule sizes larger than that of the blank formula due to the precious composition of the essential oils which increase the concentration of oil and increase droplet size. The polydispersity index (Table 3; Figure 3) did not exceed 0.41 ± 0.02 which revealed a narrow particle size distribution and homogenous droplet sizes which improves and facilitates the topical application of the formulae. The electrical surface charge or particle charge was determined by measuring the zeta potential. The values of zeta-potential for all measured samples in distilled water as a solvent is 15.8 ± 0.36 mV to 21.2 ± 0.25 mv Figure 3. The obtained results are convenient with the previously reported data which revealed that particles with surface charge ranging between −10 and +10 mV are considered neutral while between -30 mV and +30 mV are considered strongly anionic and cationic which improves sample stability (Clogston and Parti, 2011) (Table 3; Figure 3).

**TABLE 3:** Particle size and polydispersity index for blank and oil loaded formula.

Formulae	Particle Size (nm)	Polydispersity Index (PDI)	Zeta Potential (+)
F1	224.43 ± 5.77	0.34 ± 0.04	15.8 ± 0.36
F2	256.5 ± 9.04	0.23 ± 0.05	19.7 ± 0.21
F3	324.53 ± 2.41	0.41 ± 0.02	17.1 ± 0.16
F4	292.63 ± 10.86	0.35 ± 0.05	20.3 ± 0.41
F5	407.7 ± 7.27	0.36 ± 0.03	21.2 ± 0.25

### 3.3 Wound Healing Evaluation for *D. tortuosa* and *D. triradiata*

#### 3.3.1 Effect of Topical Application of *D. tortuosa* and *D. triradiata* Nanoemulsion on Wound Contraction

**FIGURE 4:**
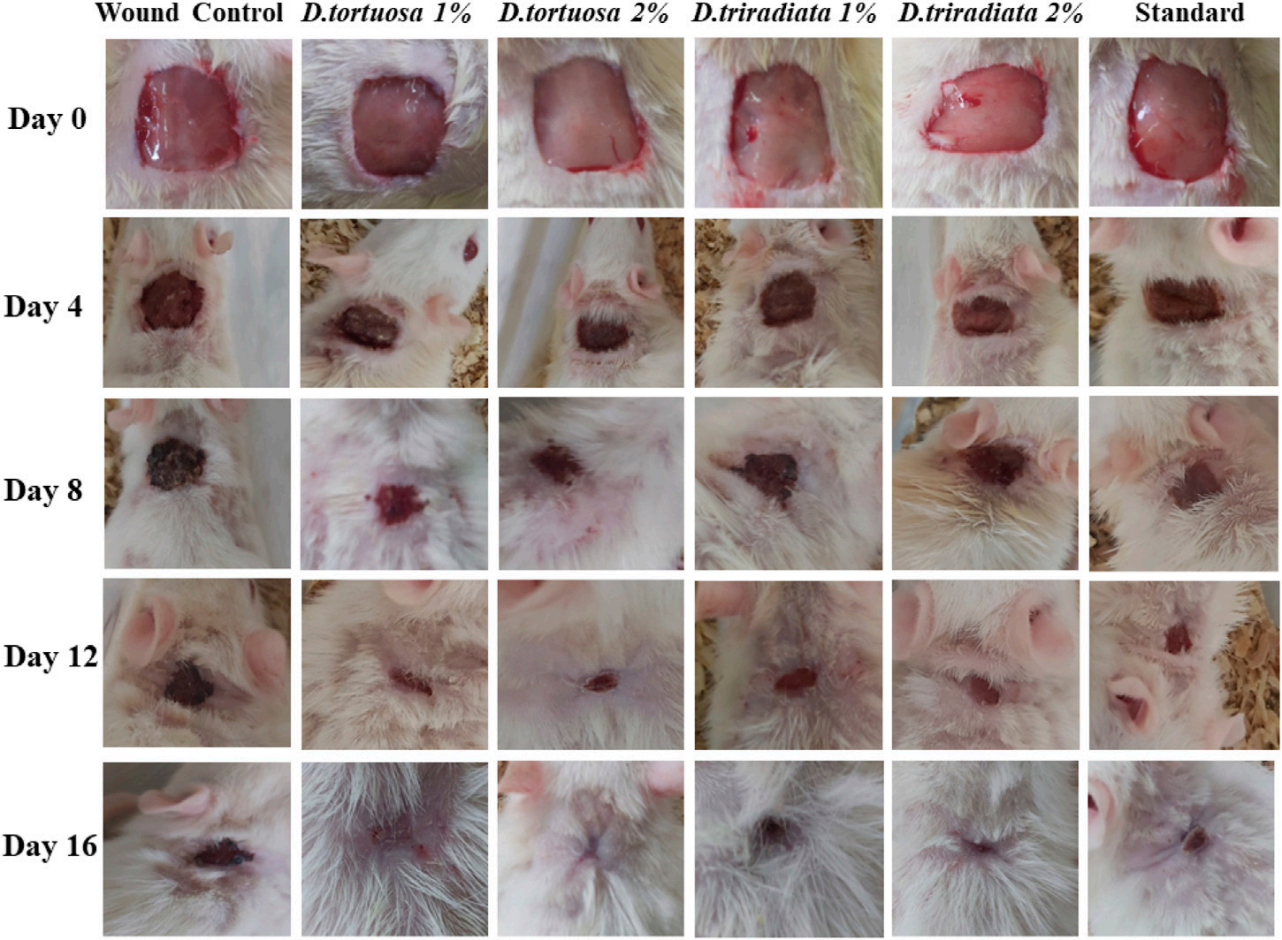
Photographic representation showing wound closure in rats treated with topical application of *D. tortuosa*, *D. triradiata* and standard on day 4, 8, 12 and 16.

**FIGURE 5:**
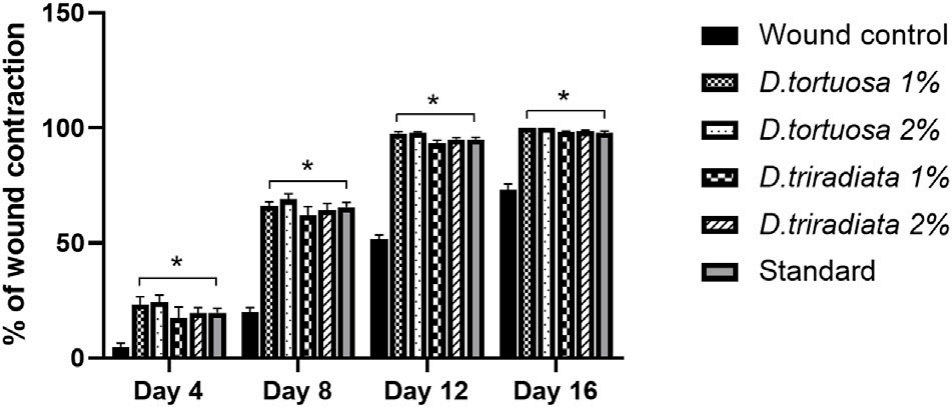
A quantitative measurement demonstrating the percentage of wound contraction in rats treated with topical application of *D. tortuosa*, *D. triradiata* and standard on day 4, 8, 12 and 16. Data presented as Mean ± SE, *n* = 6, *: significant from wound control.

Wound contraction was assessed as the percent decrease in the wound area and is indicated by observing improvement in the rate of healed area, by another means, the faster wound closure, the better drug efficacy (Prasad and Dorle, 2006). In the current study, wound contraction was calculated on the 4th, 8th, 12th, and 16th days (Figures 4 and 5). On day 4, the daily topical application of nanoemulsion of both oils and standard significantly increased the wound contraction being 23.2% and 24.4% for 1, 2% *D. tortuosa* and 17.4%, 19.5% for 1, 2% *D. triradiata* respectively, and 19.7% for the standard in comparison to the wound control group whereas on day 8 the percentage increase becomes 66, 68.9 for 1 and 2% of *D. tortuosa* respectively, 62.1, 64.2 for 1 and 2% *D. triradiata*, respectively, where the standard increase by 65.5% in comparison to the wound control group. Moreover, on the 12th the topical application of both oil formula and standard showed a better healing activity evidenced by a significant increase in the percent of wound contraction by 97.7, 98, 93.3, 95.1, and 95%, respectively, as compared to the wound control group. Notably, it was observed that *D. tortuosa* (1% or 2%) exhibited 100% wound contraction and complete healing on day 16 as well as, *D. triradiata* (1% or 2%) and standard showed 98.1, 98.6, and 97.9% of wound contraction, respectively.

#### 3.3.2 Effect of the Topical Application of *D. tortuosa* and *D. triradiata* Nanoemulsion on Lipid Peroxidation and Antioxidant Markers

**FIGURE 6:**
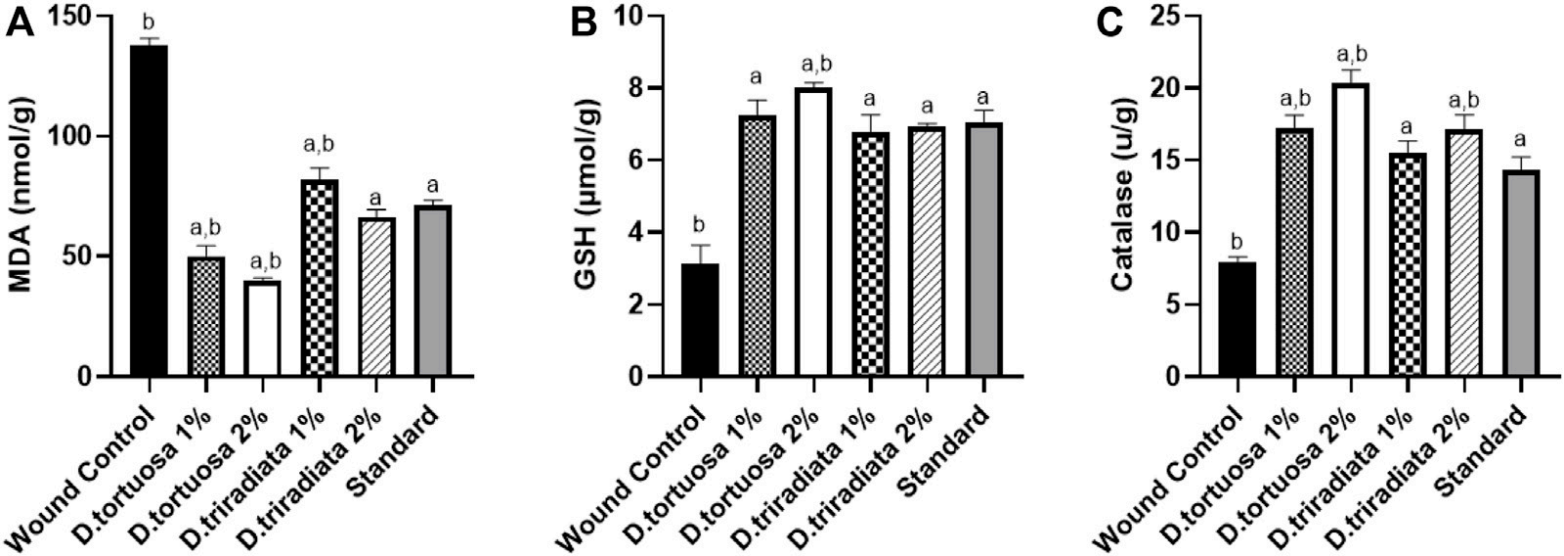
Effect of topical application of *D. tortuosa* and *D. triradiata* on lipid peroxidation and antioxidant markers. **(A)**: Lipid peroxidation level expressed as malondialdehyde (MDA), **(B)**: Reduced glutathione (GSH) and **(C)**: Catalase (CAT) in the wound tissues. Data presented as Mean ± SE, *n* = 6, a: significant from wound control, b: significant from standard.

Topical application of nanoemulsion for two oil species and the standard significantly increased the levels of GSH and CAT (*p* < 0.0001) as well as significantly decrease the MDA level (*p* < 0.0001) in skin tissues as compared to the wound control group (Figure 6). Interestingly, *D. tortuosa* 2% showed the best antioxidant effect when compared to the other treated groups*.*

#### 3.3.3 Effect of the Topical Application of *D. tortuosa* and *D. triradiata* Nanoemulsion on Inflammatory Markers

**FIGURE 7:**
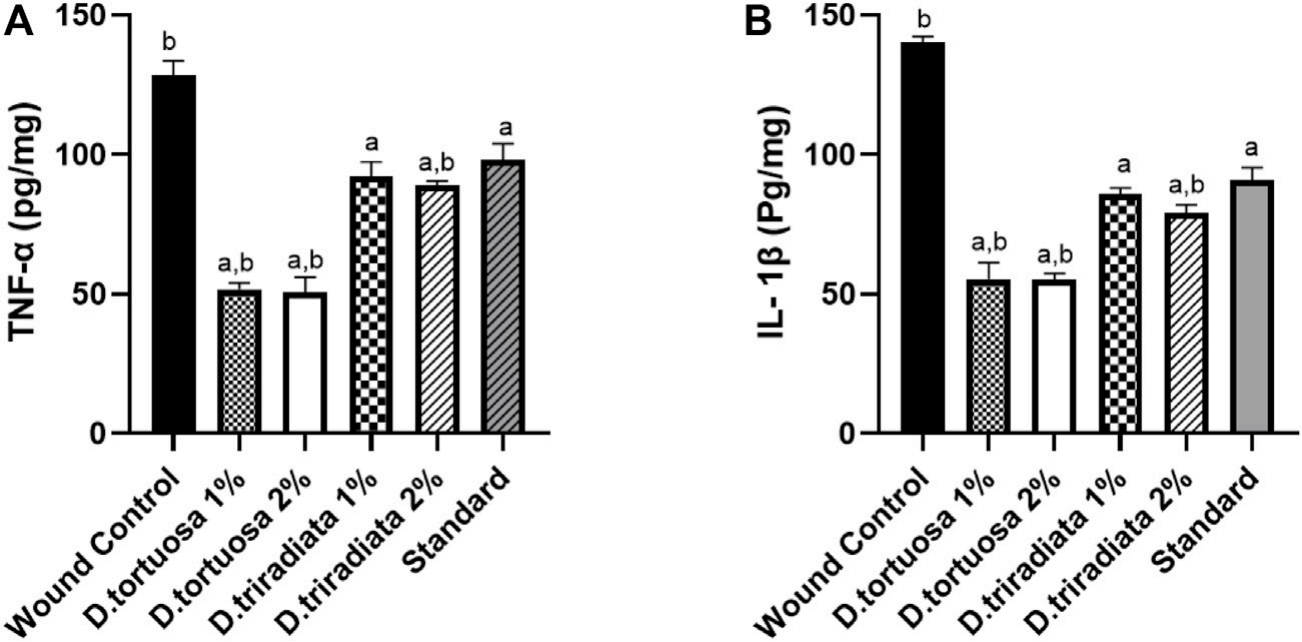
Effect of topical application of *D. tortuosa* and *D. triradiata* on inflammatory markers. **(A)**: Tumor necrosis factor α (TNF-α) and **(B)**: interleukin-1*β* (IL-1*β*) in the wound tissues. Data presented as Mean ± SE, *n* = 6, a: significant from wound control, b: significant from standard.

As shown in Figure 7, the levels of TNF-α and IL-1β decreased significantly in oil and standard groups (*p* < 0.0001) in comparison to the wound control group. Moreover, *D. tortuosa* group (2%) exhibited powerful anti-inflammatory activity in comparison to the other treated groups (Supplementary Table S3).

#### 3.3.4 Effect of Topical Application of *D. tortuosa* and *D. triradiata* on Growth Factors

**FIGURE 8:**
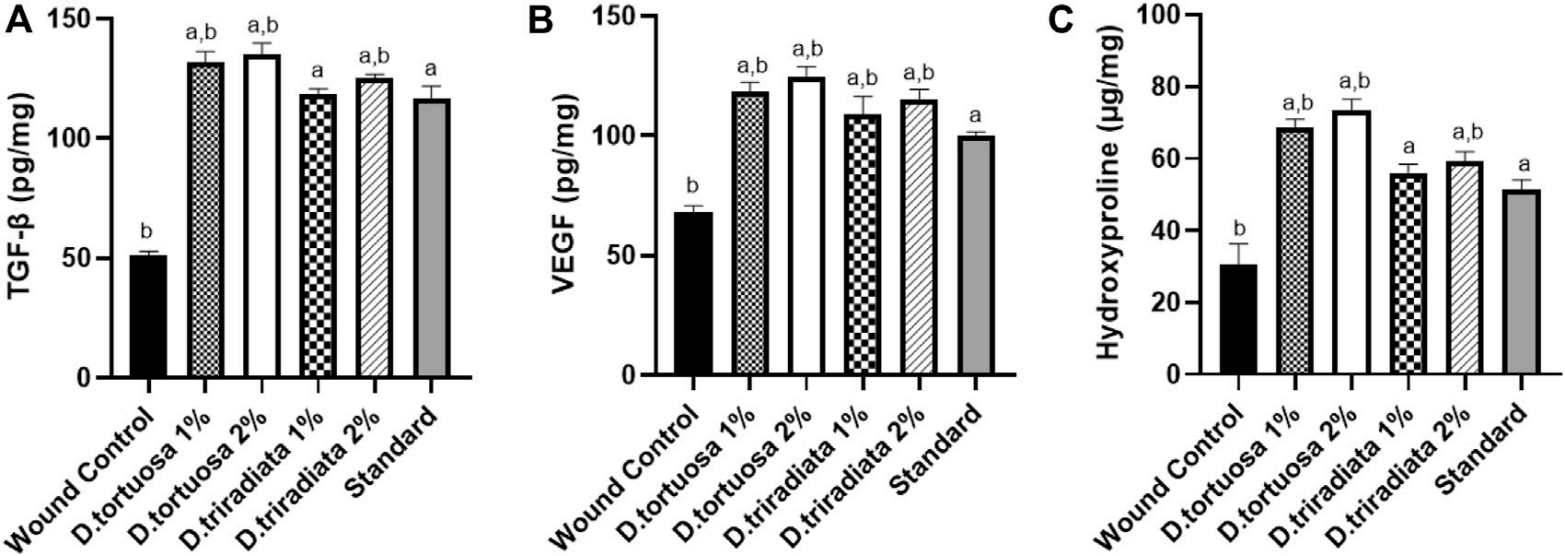
Effect of topical application of *D. tortuosa* and *D. triradiata* on growth factors and hydroxyproline content. **(A)**: Transforming growth factor beta (TGF-β), **(B)**: vascular endothelial growth factor (VEGF) and **(C)** hydroxyproline content in the wound tissues. Data presented as Mean ± SE, *n* = 6, a: significant from wound control, b: significant from standard.

Results revealed that the two oil nanoemulsions (1% or 2%) and standard groups exhibited a significant increase in TGF-β and VEGF levels (*p* < 0.0001) as compared to the wound control group (Figure 8 A&B). Moreover, *D. tortuosa* 2% group displays the highest growth factor levels. (Supplementary Table S3).

#### 3.3.5 Effect of Topical Application of *D. tortuosa* and *D. triradiata* on Hydroxyproline Content

Topical application of both *D. tortuosa* and *D. triradiata* (1% or 2%) and the standard have significantly increased the content of hydroxyproline (*p* < 0.001) in comparison to the wound control group. In addition, the highest content of hydroxyproline was observed in the case of *D. tortuosa* 2% group among other treated groups (Figure 8C) (Supplementary Table S3).

#### 3.3.6 Effect of Topical Application of *D. tortuosa* and *D. triradiata* on Histological Examination and Collagen Fibers Content

**FIGURE 9:**
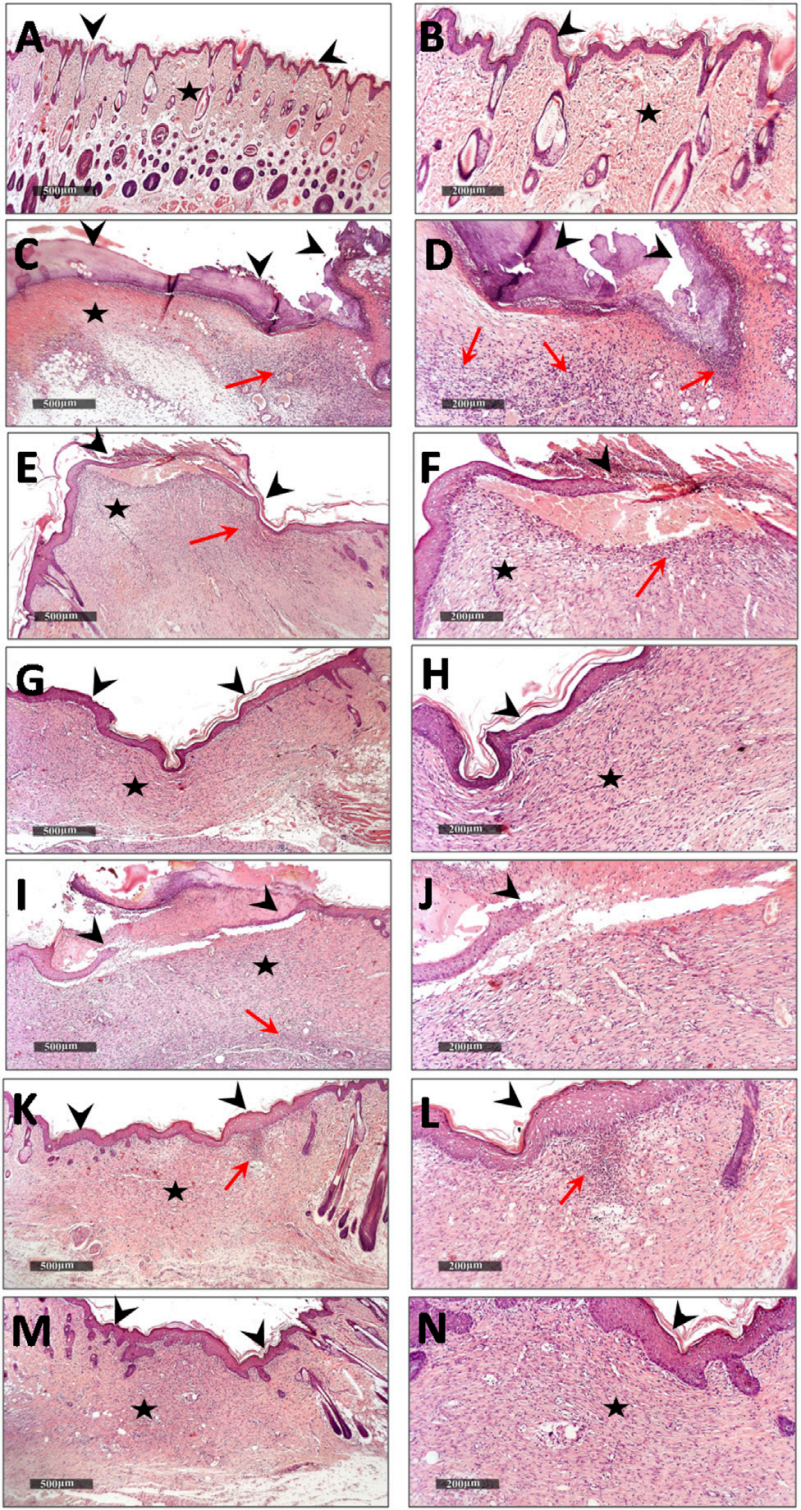
Demonstrating light microscopic histological features of skin layers and wound healing process in different groups **(A,B)**, normal control **(C,D)**, wound control group **(E,F)**. *D. tortuosa* 1% **(G,H)**, *D. tortuosa* 2%, **(I,J)**, *D. triradiata* 1%, **(K,L)**, *D. triradiata* 2% **(M,N)** and standard treated group (H&E stain). **Arrow head** **:** epidermal layer and wound gab, **Star:** skin dermis and **Red arrow:** inflammatory cells aggregates.

**FIGURE 10:**
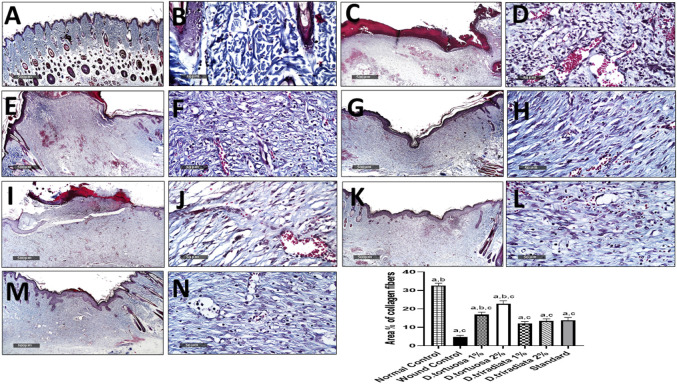
Effect of topical application of *D. tortuosa* and *D. triradiata* on collagen fibers content in dermal layer of different experimental groups **(A,B)**, normal control **(C,D)**, wound control group **(E,F)**, *D. tortuosa* 1% **(G,H)**, *D. tortuosa* 2%, **(I,J)**, *D. triradiata* 1%, **(K,L)**, *D. triradiata* 2% and standard treated group **(M,N)**. (Masson’s trichrome stain). Data presented as Mean ± SE, *n* = 6, a: significant from wound control, b: significant from standard, c: significant form normal control.

Microscopical examination for different normal skin samples revealed the well-organized morphological features of skin layers including an intact epidermal layer with intact keratinocytes covering the intact dermal layer (Figure 9A,B) with abundant records of densely packed collagen fibers up to 32.6% of dermal layer content (Figure 10A,B), many hair follicles and minimal inflammatory cell infiltrates. Intact vasculatures and subcutaneous layer were observed. In contrast, wound control group samples revealed a wide area of ulcerated wound gap covered with the scab of necrotic tissue depress and inflammatory cells, with obvious highly cellular, less fibrous newly formed granulation tissue filling the lost dermal compartment (with up to 4.8 area percentage of collagen fibers contents) (Figure 10C,D). Abundant mixed inflammatory cell infiltrates were shown all over the dermal layer with many congested subcutaneous blood vessels (Figure 9C,D). Almost the same records as wound control model samples were shown in *D. tortuosa* 1% group samples. However, the small ulcerated wound gab was still persistent (Figure 9E,F) with significantly higher dermal fibroblastic activity and collagen fibers content up to 3.5 folds compared with wounded control samples (Figure 10E,F). The most accelerated wound-healing process was demonstrated in *D. tortuosa* 2% group samples with obvious complete re-epithelialization of the epidermal layer (Figure 9G,H), and more enhanced dermal newly formed collagen fibers (up to 4.7 folds compared with wound control samples) (Figure 10G,H). Mild inflammatory cell infiltrates were observed in subepidermal and dermal layers. *D. triradiata* 1% treated samples showed almost the same records as model samples with persistent wide ulcerated wound gab and epidermal loss, as well as higher inflammatory cell, infiltrates in deep dermal and subcutaneous layers (Figure 9I,J) with only 2.4 folds’ increase in dermal collagen fibers contents (Figure 10I,J). In *D. triradiata* 2% treatment samples, although complete epidermal re-epithelialization was shown, focal subepidermal inflammatory cell aggregates were recorded (Figure 9K,L) as well as the insignificant acceleration of newly formed dermal collagen fibers were observed compared with *D. triradiata* 1% groups sample and up to 2.7 folds more than control wound samples (Figure 10K,L). Standard treated group samples showed almost the same histological features as *D. triradiata* 2% treated samples (Figure 9M,N) as well as collagen fibers contents (Figure 10M,N). Moreover; mild inflammatory cell infiltrates were observed all over the dermal layer.

Studies on the biological activity of *Deverra* species essential oil are very limited; in addition, the *in vivo* wound-healing activity of both *D. tortuosa* and *D. triradiata* essential oil was reported here for the first time.

Several reports were conducted about the essential oils' wound-healing activity using different animal models (Kumar et al., 2007; de Fatima et al., 2008; Suntar et al., 2011; Tumen et al., 2011). Moreover, many recent studies reported that when the drugs are incorporated inside the nanoemulsion core, they can penetrate easily into the skin through the subcutaneous barrier (Teichmann et al., 2007; Ahmad et al., 2018). Consequently, nanoemulsions can reduce the size of essential oil droplet to nanometer size range and therefore after applying it to the wounded sites, the water content is evaporated leaving a film on the wounded site, moreover, it protects EO components from adverse environmental conditions, and improve their stability (Perinelli et al., 2020). The colloidal system, oil-in-water nanoemulsions is considered one of the top efficient systems available for encapsulation and delivery of hydrophobic compounds, as essential oils (Cheong et al., 2018). Therefore, nanoemulsions of essential oil were prepared to improve wound-healing effects. Previous reports about the essential oil of *D. tortuosa* revealed that it exhibits antimicrobial, antioxidant, and allopathic activity (Fayed et al., 2021), and the oil of *D. triradiata* exhibits anti-inflammatory activity (Donia et al., 2015). The chemical composition of essential oil is responsible for its biological activities. As in the case of *D. tortuosa* aerial parts prepared by the SFE method revealed the presence of 20 compounds among which sabinene (20.60%) and terpinen-4-ol (19.61%) represent the major constituents while that in the case of *D. triradiata* about 18 compounds were detected in SFE sample where elemicin (34.83%) and germacrene D (23.30%) represent the major constituents. It was accepted that the restoration and functional integrity of the wound tissue comprise different biological processes such as inflammation, wound contraction, angiogenesis, and extracellular matrix deposition. Also, it was found that a single or multiple mechanisms might be responsible for different phases of wound healing which can contribute to the overall outcome of the wound-healing process (Velnar et al., 2009; Grieb et al., 2011). Inflammation and oxidative stress represent the key features that play a significant role in wound healing control. Wound healing is generally complicated due to the production of free radicals as a result of cutaneous tissue damage which may destroy lipids, proteins, and extracellular matrix (ECM) elements (Lim et al., 2018). The present study revealed that the topical application of nanoemulsion of both examined oil displays antioxidant activity, which may support the prevention of oxidative damage and upregulate the wound-healing process. Moreover, the results show that the two oils display a significant decrease in the TNF-α and IL-1β levels which leads to the inference that these were likely to be one of the factors responsible for the wound-healing process. Accordingly, they give strong evidence that the two *Deverra* oil nanoemulsions accelerated the wound-healing process. Among the major constituents of *D. tortuosa* is sabinene which is reported to exhibit significant anti-inflammatory activity through the inhibition of pro-inflammatory cytokines, TNF-α, IL-1β, and -6 (Valente et al., 2009) and inhibition of NO production in macrophages (Valente et al., 2009). Also, it was reported that terpinen-4-ol exhibits anti-inflammatory activity through the suppression of TNF-α, IL-1β, and IL-10 factors (Hart et al., 2000) and it represents a major component of much essential oil which is used as an anti-inflammatory and antioxidant agent (Shapira et al., .2016). Moreover, it was found that the major compounds present in *D. triradiata* is related to phenylpropanoids possess antimicrobial and antioxidant activities, and may help in the healing process (Korkina, 2007). It was found that elemicin (da Silveira et al., 2014; Ilijeva and Buchbauer, 2016) and germacrene D (Bayala et al., 2014; Sitarek et al., 2017) possess anti-inflammatory and antioxidant activities. Moreover, it was reported that there is also the possibility of a synergistic effect between the different compounds in essential oils instead of only one major compound or isolated substances acting on the healing pathways. It was documented that fibroblasts have an essential role in the synthesis of collagen fibers, regeneration of extracellular matrix, and the release of endogenous growth factors such as TGF-β and VEGF helping in re-epithelization and remodeling of wounds (Werner and Grose, 2003). Collagen is one of the predominant components of the extracellular matrix related to the process of wound healing (Soliman et al., 2018). In the current study, the significantly elevated levels of collagen and hydroxyproline in rats treated with *D. tortuosa* or *D. triradiata* demonstrate their beneficial effects on the process of wound closure and complete wound healing. Degradation of collagen leads to the liberation of free hydroxyproline, which is used as an indicator for collagen turnover leading to rapid healing. Moreover, the wound-healing process is confirmed by high levels of TGF-β and VEGF in wound tissues.

## 4 Conclusion

Due to the extent and increasing frequency of different types of skin injuries, skin regeneration is a challenge that needs close collaboration between researchers in many disciplines. Our study will lead to enhanced wound healing treatments in the term of topical application of two *Deverra* species essential oils in the form of nanoemulsion formulation. The nanoemulsion exhibits significant antioxidants and anti-inflammatory effects and, displays a significant increase in growth factors and hydroxyproline levels and demonstrated complete re-epithelialization associated with activated hair follicles and abundant collagen fibers.

## Data Availability

The original contributions presented in the study are included in the article/Supplementary Material, further inquiries can be directed to the corresponding authors.

## References

[B1] Abbas H. El Sayed N. S. Ali M. E. Elsheikh M. A. **(** 2021 **).** Integrated Lecithin–Bile Salt Nanovesicles as a Promising Approach for Effective Skin Delivery of Luteolin to Improve UV-Induced Skin Damage in Wistar Albino Rats. Colloids Surf. B Biointerfaces. 2211, 112299. ‏ 10.1016/j.colsurfb.2021.112299 34953364

[B2] Abdallah H. M. Ezzat S. M. (2011). Effect of the Method of Preparation on the Composition and Cytotoxic Activity of the Essential Oil of *Pituranthos Tortuosus* . Z Naturforsch C J. Biosci. 66, 143–148. 10.1515/znc-2011-3-408 21630588

[B3] Abdel-Ghani A. Hafez S. S. (1995). GC-MS Analysis and Antimicrobial Activity of Essential Oil of *Pituranthos Tortuosus* (Desf.). Qatar Univ. Sci. J. 15, 23–26.

[B4] Adams R. P. (2005). Identification of Essential Oil Components by Gas Chromatography/quadrupole Mass Spectroscopy. J. Am. Soc. Mass Spectrom. 16, 1902–1903.

[B5] Agha R. Ogawa R. Pietramaggiori G. Orgill D. P. (2011). A Review of the Role of Mechanical Forces in Cutaneous Wound Healing. J. Surg. Res. 171, 700–708. 10.1016/j.jss.2011.07.007 22005503

[B80] Ahmad N. Alam Md. A. Ahmad F. J. Sarafroz Md. Ansari K. Sharma S. (2018). Ultrasonication Techniques Used for the Preparation of Novel Eugenol–Nanoemulsion in the Treatment of Wounds Healings and Anti-Inflammatory. J. Drug. Delivery. Sci. Technol. 46, 461–473. 10.1016/j.jddst.2018.06.003

[B6] Alam P. Shakeel F. Anwer M. K. Foudah A. I. Alqarni M. H. (2018). Wound Healing Study of eucalyptus Essential Oil Containing Nanoemulsion in Rat Model. J. Oleo Sci. 67 (8), 957–968. 10.5650/jos.ess18005 30012898

[B7] Ali S. M. Yosipovitch G. (2013). Skin pH: From Basic Science to Basic Skin Care. Acta Derm. Venereol. 93, 261–267. 10.2340/00015555-1531 23322028

[B8] Ashkenazy D. Friedman J. Kashman Y. (1983). The Furocoumarin Composition of Pituranthos Triradiatus. Planta Med. 47, 218–220. 10.1055/s-2007-969990 17404918

[B9] Aswathanarayan J. B. Vittal R. R. (2019). Nanoemulsions and Their Potential Applications in Food Industry. Front. Sustain. Food Syst. 3, 1–21. 10.3389/fsufs.2019.00095

[B10] Bayala B. Bassole I. H. N. Gnoula C. Nebie R. Yonli A. Morel L. (2014). Chemical Composition, Antioxidant, Anti-inflammatory and Anti-proliferative Activities of Essential Oils of Plants from *Burkina Faso* . PloS one 9, e92122. 10.1371/journal.pone.0092122 24662935 PMC3963878

[B11] Boulos L. (2000). Flora of Egypt, 2. Cairo: AL-Hadara Publishing.

[B12] Carvalho Filho J. L. S. Blank A. F. Alves P. B. Ehlert P. A. Melo A. S. Cavalcanti S. C. (2006). Influence of the Harvesting Time, Temperature and Drying Period on Basil (*Ocimum Basilicum* L.) Essential Oil. Rev. Bras. Farmacogn. 16, 24–30. 10.1590/s0102-695x2006000100007

[B13] Chaduhari P. M. Kuchekar M. A. (2018). Development and Evaluation of Nanoemulsion as a Carrier for Topical Delivery System by Box-Behnken Design. Asian J. Pharm. Clin. Res. 11, 286–293. 10.22159/ajpcr.2018.v11i8.26359

[B14] Cheong A. M. Tan C. P. Nyam K. L. (2018). Effect of Emulsification Method and Particle Size on the Rate of *In Vivo* Oral Bioavailability of Kenaf (*Hibiscus Cannabinus* L.) Seed Oil. J. Food. Sci. 83, 1964–1969. 10.1111/1750-3841.14191 29802733

[B15] Clogston J. D. Patri A. K. (2011). Zeta Potential Measurement: Characterization of Nanoparticles Intended for Drug Delivery. Methods Mol. Biol. Clift. N.J.) 697, 63–70. 10.1007/978-1-60327-198-1_6 21116954

[B16] Culling C. F. A. (2013). Handbook of Histopathological and Histochemical Techniques: Including Museum Techniques (London, UK: Butterworths), 3.

[B17] da Silveira E. Sá. R. C. Andrade L. N. de Oliveira R. B. de Sousa D. P. (2014). A Review on Anti-inflammatory Activity of Phenylpropanoids Found in Essential Oils. Molecules 19, 1459–1480. 10.3390/molecules19021459 24473208 PMC6270723

[B18] Davoodi-Roodbordeii F. Afshar M. Haji Abas T. F. Choopani S. Torkaman G. Moayer F. (2019). Topical Hydrogel Containing *Fumaria Vaillantii* Loisel. Extract Enhances Wound Healing in Rats. BMC Complement. Altern. Med. 19, 254. 10.1186/s12906-019-2645-y 31511001 PMC6739951

[B19] de Fatima A. Modolo L. V. Sanches A. C. Porto R. R. (2008). Wound Healing Agents: the Role of Natural and Non-nat Ural Products in Drug Development. Mini Rev. Med. Chem. 8, 879–888. 10.2174/138955708785132738 18691145

[B20] Denys D. J. Simon J. E. (1990). Comparison of Extraction Methods for the Rapid Determination of Essential Oil Content and Composition of Basil. J. Amer. Soc. Hort. Sci. 115 (3), 458–462.

[B21] Diegelmann R. F. Evans M. C. (2004). Wound Healing: An Overview of Acute, Fibrotic and Delayed Healing. Front. Biosci. 9, 283–289. 10.2741/1184 14766366

[B22] Donia A. M. Soliman G. A. Al-Saikhan F. I. Gabr G. A. Ganaie M. A. Ansari M. N. (2015). The Potential Anti-inflammatory Activity of Essential Oils of *Pituranthos Triradiatus* and *Anthemis Deserti* in Rats. BEPLS 4, 28–31.

[B23] Elmesallamy A. M. D. Mohamed E. I. Sarhan M. A. M. Hussein S. A. M. (2021). Chemical and Biological Activities of *Deverra Triradiata* Hochst. Ex. Boiss. Aerial Parts from St.Catherine, Southern Sinai, Egypt. Egypt. Egypt. J. Chem. 64, 1387–1394. 10.21608/ejchem.2020.52846.3092

[B24] Elshibani F. Alshalmani S. Mohammed H. A. (2020). *Pituranthos Tortuosus* Essential Oil from Libya: Season Effect on the Composition and Antioxidant Activity. Essent. Oil-Bear. Plants. 23, 1095–1104. 10.1080/0972060X.2020.1843550

[B25] Enoch S. Leaper D. J. (2008). Basic Science of Wound Healing. Surgery 26, 31–37. 10.1016/j.mpsur.2007.11.005

[B26] Fayed E. M. Abd‐EIGawad A. M. Elshamy A. I. El‐Halawany E. F. Ei‐Amier Y. A. (2021). Essential Oil of *Deverra Tortuosa* Aerial Parts: Detailed Chemical Profile, Allelopathic, Antimicrobial, and Antioxidant Activities. Chem. Biodivers. 18 (4), 1–14. 10.1002/cbdv.202000914 33606911

[B27] Gad H. Al-Sayed E. Ayoub I. (2021). Phytochemical Discrimination of Pinus Species Based on GC–MS and ATR-IR Analyses and Their Impact on *Helicobacter pylori* . Phytochem. Anal. 32, 1–16. 10.1002/pca.3028 33462938

[B28] Ghazanfar N. Mortazavi A. S. Yazdi T. S. Mohammadi M. (2020). Microwave-assisted Hydrodistillation Extraction of Essential Oil from Coriander Seeds and Evaluation of Their Composition, Antioxidant and Antimicrobial Activity. Heliyon 6, e048932. 10.1016/j.heliyon.2020.e04893 PMC7498746 32984601

[B29] Grieb G. Steffens G. Pallua N. Bernhagen J. Bucala R. (2011). Circulating Fibrocytes-Biology, and Mechanisms in Wound Healing and Scar Formation. Int. Rev. Cell Mol. Biol. 291, 1–19. 10.1016/B978-0-12-386035-4.00001-X 22017972

[B30] Guetat A. (2022). The Genus Deverra DC. (Syn. Pituranthos Viv.): A Natural Valuable Source of Bioactive Phytochemicals: A Review of Traditional Uses, Phytochemistry and Pharmacological Properties. J. Ethnopharmacol. 284, 114447. 10.1016/j.jep.2021.114447 34737008

[B31] Gurtner G. C. Werner S. Barrandon Y. Longaker M. T. (2008). Wound Repair and Regeneration. Nature 453, 314–321. 10.1038/nature07039 18480812

[B32] Halim A. F. Lahloub M. F. I. Saad H-E. A. Ahmed A. F. (1991). Coumarins of Roots of *Pituranthos Triradiatus* Growing in Egypt. MJPS 7 (3), 402–413.

[B33] Hamdan S. Pastar I. Drakulich S. Dikici E. Tomic-Canic M. Deo S. (2017). Nanotechnology-driven Therapeutic Interventions in Wound Healing: Potential Uses and Applications. ACS Cent. Sci. 3, 163–175. 10.1021/acscentsci.6b00371 28386594 PMC5364456

[B34] Hart P. H. Brand1 C. F. Carson T. V. Riley R. H. Prager R. H. Finlay-Jones 1. J. J. (2000). Terpinen-4-ol, the Main Component of the Essential Oil of Melaleuca Alternifolia (Tea Tree Oil), Suppresses Inflammatory Mediator Production by Activated Human Monocytes. Inflamm. Res. 49, 619–626. 10.1007/s000110050639 11131302

[B35] Ilijeva R. Buchbauer G. (2016). Biological Properties of Some Volatile Phenylpropanoids. Nat. Nat. Prod. Commun. 1 (10), 1619–1629. 10.1177/1934578x1601101041 30549629

[B36] Koedam A. Scheffer J. C. S. Svendsen A. B. (1979). Comparison of Isolation Procedures for Essential Oils. ZLUF 168, 106–111. 10.1007/BF01127514

[B37] Korkina L. G. (2007). Phenylpropanoids as Naturally Occurring Antioxidants: from Plant Defense to Human Health. Cell Mol. Biol. 53, 15–25. 10.1170/T772 17519109

[B38] Koshak A. E. Algandaby M. M. Mujallid M. I. Abdel-Naim A. B. Alhakamy N. A. Fahmy U. A. (2021). Wound Healing Activity of Opuntia *Ficus-Indica* Fixed Oil Formulated in a Self-Nanoemulsifying Formulation. Int. J. nanomedicine 16, 3889–3905. 10.2147/IJN.S299696 34135583 PMC8200171

[B39] Krifa M. Meshri S. E. E. Bentouati N. Pizzi A. Sick E. Chekir-Ghedira L. (2016). *In Vitro* and *In Vivo* Anti-melanoma Effects of *Pituranthos Tortuosus* Essential Oil *via* Inhibition of FAK and Src Activities. J. Cell. Biochem. 117 (5), 1167–1175. 10.1002/jcb.25400 26477879

[B40] Kumar B. Vijayakumar M. Govindarajan R. Pushpangadan P. (2007). Ethnopharmacological Approaches to Wound Healing Exploring Medicinal Plants of India. J. Ethnopharmacol. 114, 103–113. 10.1016/j.jep.2007.08.010 17884316

[B41] Kundu A. Ghosh A. Singh N. K. Singh G. K. Seth A. Maurya S. K. (2016). Wound Healing Activity of the Ethanol Root Extract and Polyphenolic Rich Fraction from Potentilla Fulgens. Pharm. Biol. 54, 2383–2393. 10.3109/13880209.2016.1157192 27043472

[B42] Labib R. M. Ayoub I. M. Michel I, H. H. Mehanny M. Kamil V. Hany M. (2019). Appraisal on the Wound Healing Potential of *Melaleuca Alternifolia* and *Rosmarinus Officinalis* L. Essential Oil-Loaded Chitosan Topical Preparations. PLOSone 14, e0219561. 10.1371/journal.pone.0219561 PMC6746351 31525200

[B43] Lim J. H. Lee B. Y. Kim J. W. Han Y. J. Chung J. H. Kim M. H. (2018). Evaluation of Extraction Methods for Methylated Cell Free Fetal DNA from Maternal Plasma. J. Assist. Reprod. Genet. 35 (4), 637–641. 10.1007/s10815-018-1114-8 29423788 PMC5949107

[B44] Mahran G. H. Ahmed M. S. Seida A. A. Amarquaye A. A. (1989). A Phytochemical Investigation of *Pituranthos Tortuosus* (Desf.) Benth and Hook. Bull. Fac. Pharm. Cairo Univ. 27, 87–89.

[B45] Martin P. Leibovich S. J. (2005). Inflammatory Cells during Wound Repair: The Good, the Bad and the Ugly. Trends Cell Biol. 15, 599–607. 10.1016/j.tcb.2005.09.002 16202600

[B46] Meyer-Warnod B. (1984). Natural Essential Oils: Extraction Processes and Application to Some Major Oils. P&F. 9, 93–104.

[B47] Miguel M. G. (2010). Antioxidant and Anti-Inflammatory Activities of Essential Oils: A Short Review. Molecules 15, 9252–9287. 10.3390/molecules15129252 21160452 PMC6259136

[B48] Mishra S. Rathore A. K. (2021). Comparative Study of the Performance of Supercritical Fluid Extraction, Microwave Assisted Hydro-Distillation and Hydro-Distillation of Lemongrass (*Cymbopogon Citratus)*: A Review. G- J. Environ. Sci. Technol. 8 (2), 20–27.

[B49] Mostafa M. Amer N. Serag M. Khedr A-H. Abdel-Mogib M. (2020). Phytochemical Constituents and Antibacterial Activity of the Medicinal Herb *Deverra Tortuosa* (Desf.) DC. RJPBCS 11, 108–115. 10.33887/rjpbcs/2020.11.2.13

[B50] Orchard A. van Vuuren S. (2017). Commercial Essential Oils as Potential Antimicrobials to Treat Skin Diseases. Evid. Based Altern. Med. 2017, 4517971. 10.1155/2017/4517971 PMC5435909 28546822

[B51] Patel R. P. Singh R. Rao B. R. Singh R. Srivastava A. Lal R. (2016). Differential Response of Genotype× Environment on Phenology, Essential Oil Yield and Quality of Natural Aroma Chemicals of Five *Ocimum* Species. Ind. Crops Prod. 87, 210–217. 10.1016/j.indcrop.2016.04.001

[B52] Perinelli D. R. Palmieri G. F. Cespi M. Bonacucina G. (2020). Encapsulation of Flavours and Fragrances into Polymeric Capsules and Cyclodextrins Inclusion Complexes: An Update. Molecules 25 (24), 5878. 10.3390/molecules25245878 PMC7763935 33322621

[B53] Prasad V. Dorle A. K. (2006). Evaluation of Ghee Based Formulation for Wound Healing Activity. J. Ethnopharmacol. 107, 38–47. 10.1016/j.jep.2006.02.006 16546334

[B54] Rassem H. H. A. Nour A. H. Yunus R. M. (2016). Techniques for Extraction of Essential Oils from Plants: A Review. A Review. Aust. J. Basic & Appl. Sci. 10 (16), 117–127.

[B55] Sargenti S. Lancas F. N. (1997). Supercritical Fluid Extraction of *Cymbopogon Citratus* (DC. Stapf. Chromatogr. 46, 285–290. 10.1007/BF02496320

[B56] Schreml S. Szeimies R. M. Prantl L. Karrer S. Landthaler M. Babilas P. (2010). Oxygen in Acute and Chronic Wound Healing. Br. J. Dermatol. 163, 257–268. 10.1111/j.1365-2133.2010.09804.x 20394633

[B57] Shakeel F. Baboota S. Ahuja A. Ali J. Shafiq S. (2008). Skin Permeation Mechanism and Bioavailability Enhancement of Celecoxib from Transdermally Applied Nanoemulsion. J. Nanobiotechnology 6, 8. 10.1186/1477-3155-6-8 18613981 PMC2481266

[B58] Shapira S. Pleban S. Kazanov D. Tirosh P. Arbe N. (2016). Terpinen-4-ol: A Novel and Promising Therapeutic Agent for Human Gastrointestinal Cancers. PLOS ONE 11, e0156540. 10.1371/journal.pone.0156540 27275783 PMC4898785

[B59] Shukla T. Upmanyu N. Agrawal M. Saraf S. Saraf S. Alexander A. (2018). Biomedical Applications of Microemulsion through Dermal and Transdermal Route. Biomed. Pharmacother. 108, 1477–1494. 10.1016/j.biopha.2018.10.021 30372850

[B60] Silva L. L. Garlet Q. I. Benovit S. C. Dolci G. Mallmann C. A. Bürger M. E. (2013). Sedative and Anesthetic Activities of the Essential Oils of *Hyptis Mutabilis* (Rich.) Briq. And Their Isolated Components in Silver Catfish (*Rhamdia quelen*). Braz. J. Med. Biol. Res. 46, 771–779. 10.1590/1414-431X20133013 24068193 PMC3854430

[B61] Singab A-N. Khalifa T. Mahran G. H. Okada Y. Matsumaru Y. Masuda N. (1998). A New Flavonoid Glycoside from *Pituranthos Tortuosus* Desf, Benth & Hook. J. Nat. Med. 52 (2), 191–194.

[B62] Singab A. B. (2003). Essential Oils and Lipids Content of Pituranthos Species Growing in Egypt. Bull. Fac. Pharm. Cairo Univ. 41, 213–217.

[B63] Singh M. R. Saraf S. Vyas A. Jain V. Singh D. (2013). Innovative Approaches in Wound Healing: Trajectory and Advances. Artif. Cells Nanomed. Biotechnol. 41, 202–212. 10.3109/21691401.2012.716065 23316788

[B64] Sitarek P. Rijo P. Garcia C. Skała E. Kalemba D. Białas J. (2017). A Antibacterial, Anti-inflammatory, Antioxidant, and Antiproliferative Properties of Essential Oils from Hairy and Normal Roots of *Leonurus Sibiricus* L. And Their Chemical Composition. Oxid. Mede Cell Longev. 2017, 7384061. 10.1155/2017/7384061 PMC5278227 28191277

[B65] Soliman A. M. Lin T. S. Ghafar N. A. Das S. (2018). Virgin Coconut Oil and Diabetic Wound Healing: Histopathological and Biochemical Analysis. Eur. J. Anat. 22 (2), 135–144.

[B66] Sorg H. Tilkorn D. J. Hager S. Hauser J. Mirastschijski U. (2017). Skin Wound Healing: An Update on the Current Knowledge and Concepts. Eur. Surg. Res. 58, 81–94. 10.1159/000454919.6 27974711

[B67] Suetsugu T. Tanaka M. Iwai H. Matsubara T. Kawamoto Y. Saito C. (2013). Supercritical CO2 Extraction of Essential Oil from Kabosu (Citrus Sphaerocarpa Tanaka) Peel. Flavour 2, 18. 10.1186/2044-7248-2-18

[B68] Suntar I. Akkol E. K. Keles H. Oktem A. Baser K. H. Yesilada E. (2011). A Novel Wound Healing Ointment: a Formulation of *Hypericum perforatum* Oil and Sage and Oregano Essential Oils Based on Traditional Turkish. Knowledge. J. Ethnopharmacol. 134, 89–96. 10.1016/j.jep.2010.11.061 21130859

[B69] Täckholm V. (1974). Student Flora of Egypt. Beirut: Pub. Cairo University. Printed by Cooperative Printing Co.

[B70] Tarnuzzer R. W. Schultz G. S. (1996). Biochemical Analysis of Acute and Chronic Wound Environments. Wound Repair Regen. 4, 321–325. 10.1046/j.1524-475X.1996.40307.x 17177727

[B71] Teichmann A. S. Heuschkel U. Jacobi G. Presse R. H. Neubert W. Sterry L. J. (2007). Comparison of Stratum Corneum Penetration and Localization of a Lipophilic Model Drug Applied in an O/w Microemulsion and an Amphiphilic Cream. Eur. J. Pharm. Biopharm. 67 (3), 699–706. 10.1016/j.ejpb.2007.04.006 17537622

[B72] Thakur R. Jain N. Pathak R. Sandhu S. S. (2007). Practices in Wound Healing Studies of Plants. Evid. Based Complement. Altern. Med. 2011, 438056. 10.1155/2011/438056 PMC3118986 21716711

[B73] Tumen I. Akkol E. K. Suntar I. Keles H. (2011). Wound Repair and Anti-inflammatory Potential of Essential Oils from Cones of Pinaceae: Preclinical Experimental Research in Animal Models. J. Ethnopharmacol. 137, 1215–1220. 10.1016/j.jep.2011.07.046 21816214

[B74] Valente J. M. Zuzarte M. J. Gonçalves M. C. Lopes C. Cavaleiro L. Salgueiro M. T. (2009). Antifungal, Antioxidant and Anti-inflammatory Activities of *Oenanthe Crocata* L. Essential Oil. FCT 62, 349–354. 10.1016/j.fct.2013.08.083 24012643

[B75] Velnar T. Bailey T. Smrkolj V. (2009). The Wound Healing Process: An Overview of the Cellular and Molecular Mechanisms. J. Int. Med. Res. 37, 1528–1542. 10.1177/147323000903700531 19930861

[B76] Vérité P. Nacer A. Kabouche Z. Seguin E. (2004). Composition of Seeds and Stems Essential Oils of *Pituranthos Scoparius* (Coss. Dur.) Schinz. lavour Fragr. J. 19, 562–564. 10.1002/ffj.1353

[B77] Wang P. H. Huang B. S. Horng H. C. Yeh C. C. Chen Y. J. (2018). Wound Healing. J. Chin. Med. Assoc. 81, 94–101. 10.1016/j.jcma.2017.11.002 29169897

[B78] Werner S. Grose R. (2003). Regulation of Wound Healing by Growth Factors and Cytokines. Physiol. Rev. 83, 835–870. 10.1152/physrev.2003.83.3.835 12843410

[B79] Wilgus T. A. (2008). Immune Cells in the Healing Skin Wound: Influential Players at Each Stage of Repair. Pharmacol. Res. 58, 112–116. 10.1016/j.phrs.2008.07.009 18723091

