# Supplementary figures and images for: Appraisal on the Wound Healing Potential of Deverra tortuosa DC. and Deverra triradiata Hochst Essential Oil Nanoemulsion Topical Preparation
Source: Front Pharmacol. 2022 Jul 26;13:940988. doi: 10.3389/fphar.2022.940988 (PMC9360601; doi:10.3389/fphar.2022.940988)

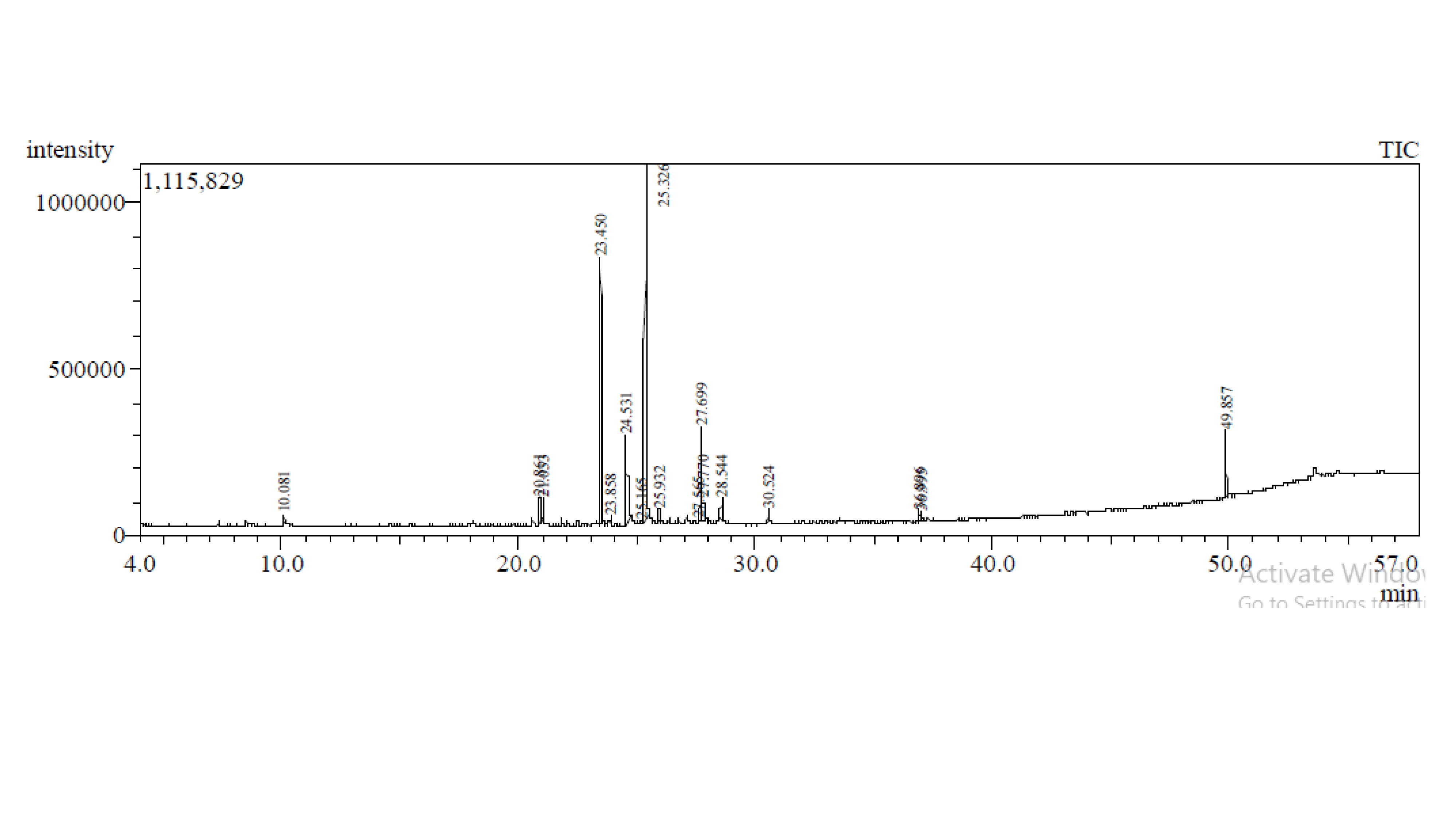

Supplement: Supplementary file 1 [file Image6.TIF]

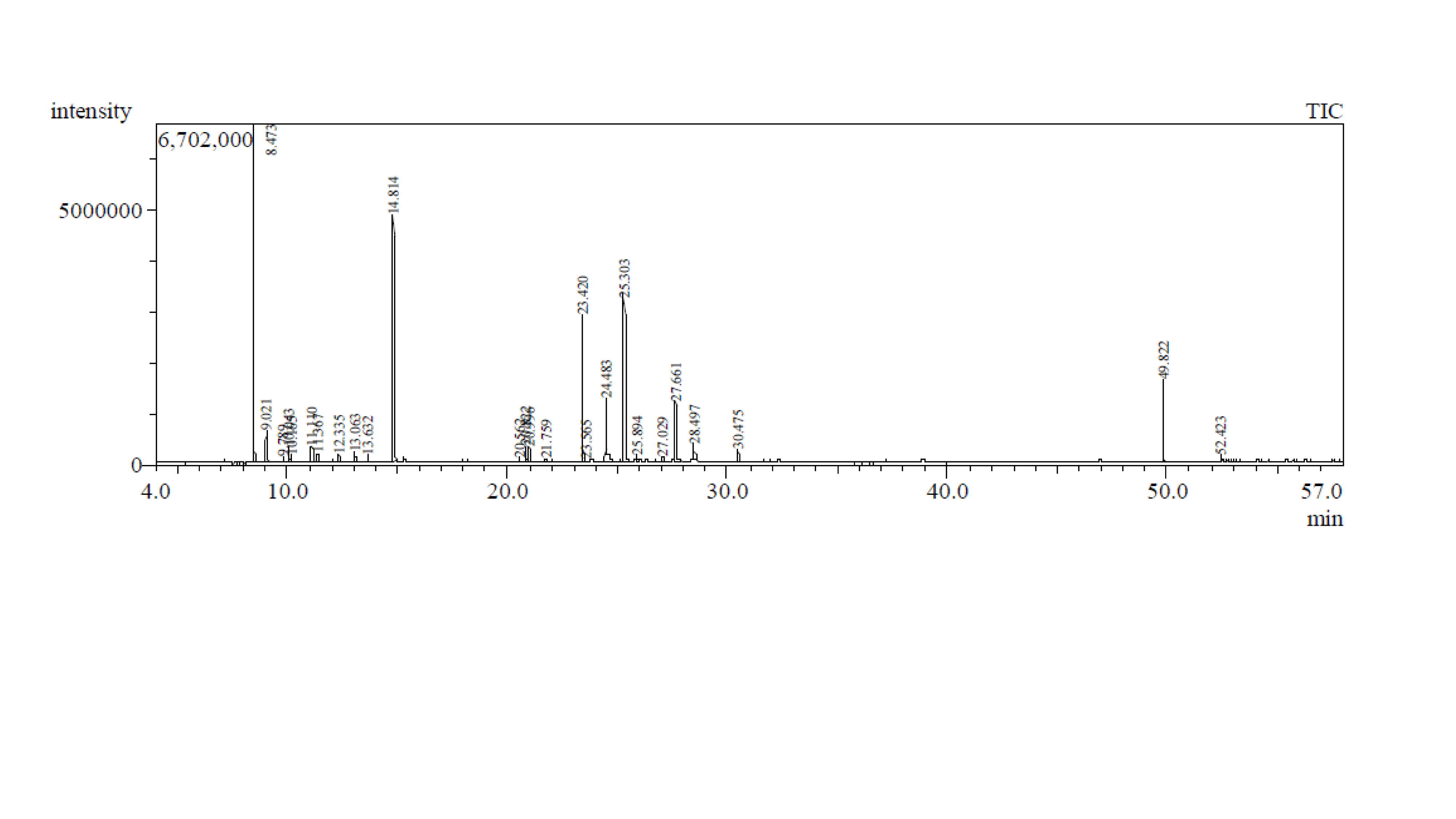

Supplement: Supplementary file 2 [file Image3.TIF]

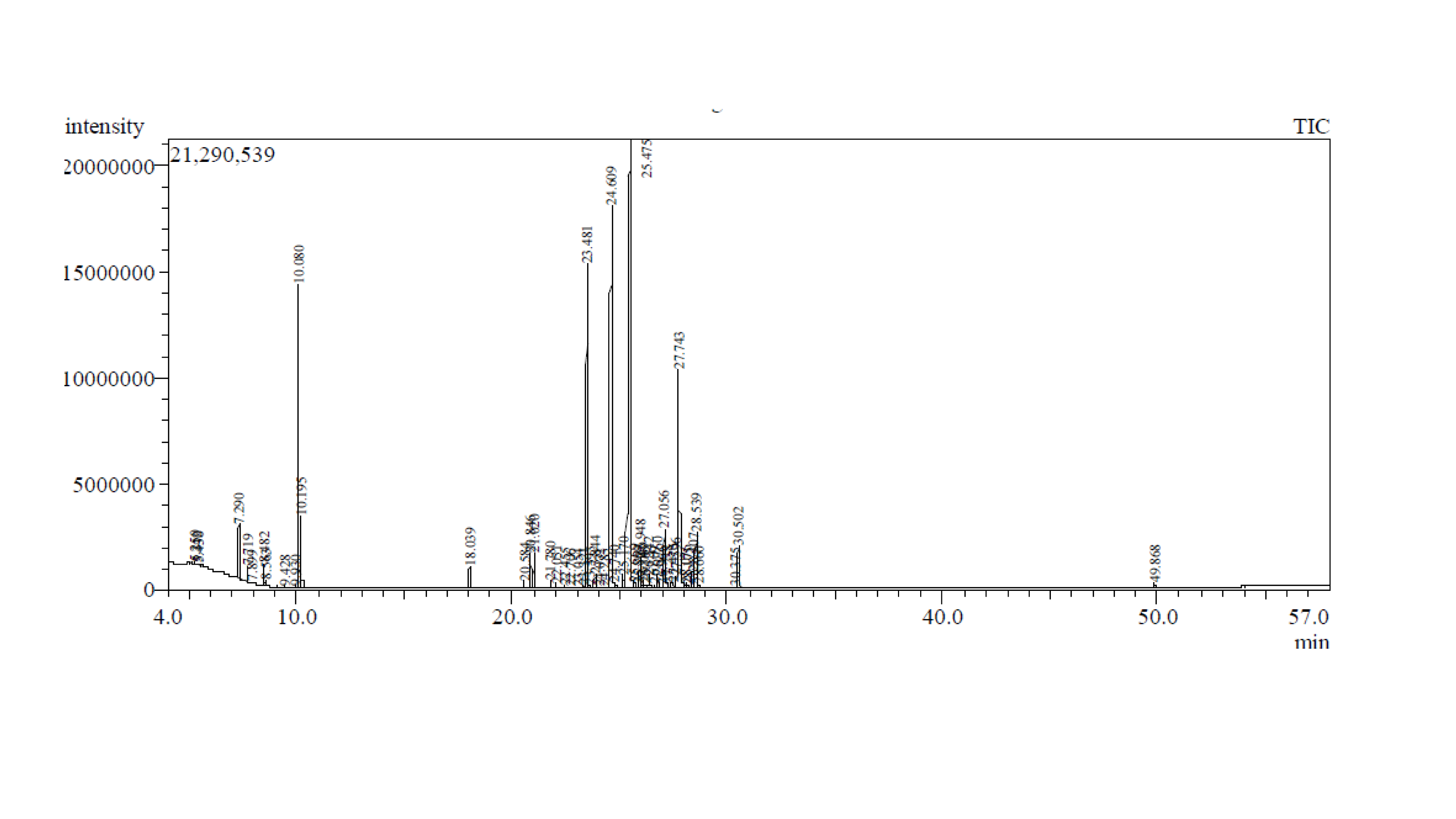

Supplement: Supplementary file 3 [file Image4.TIF]

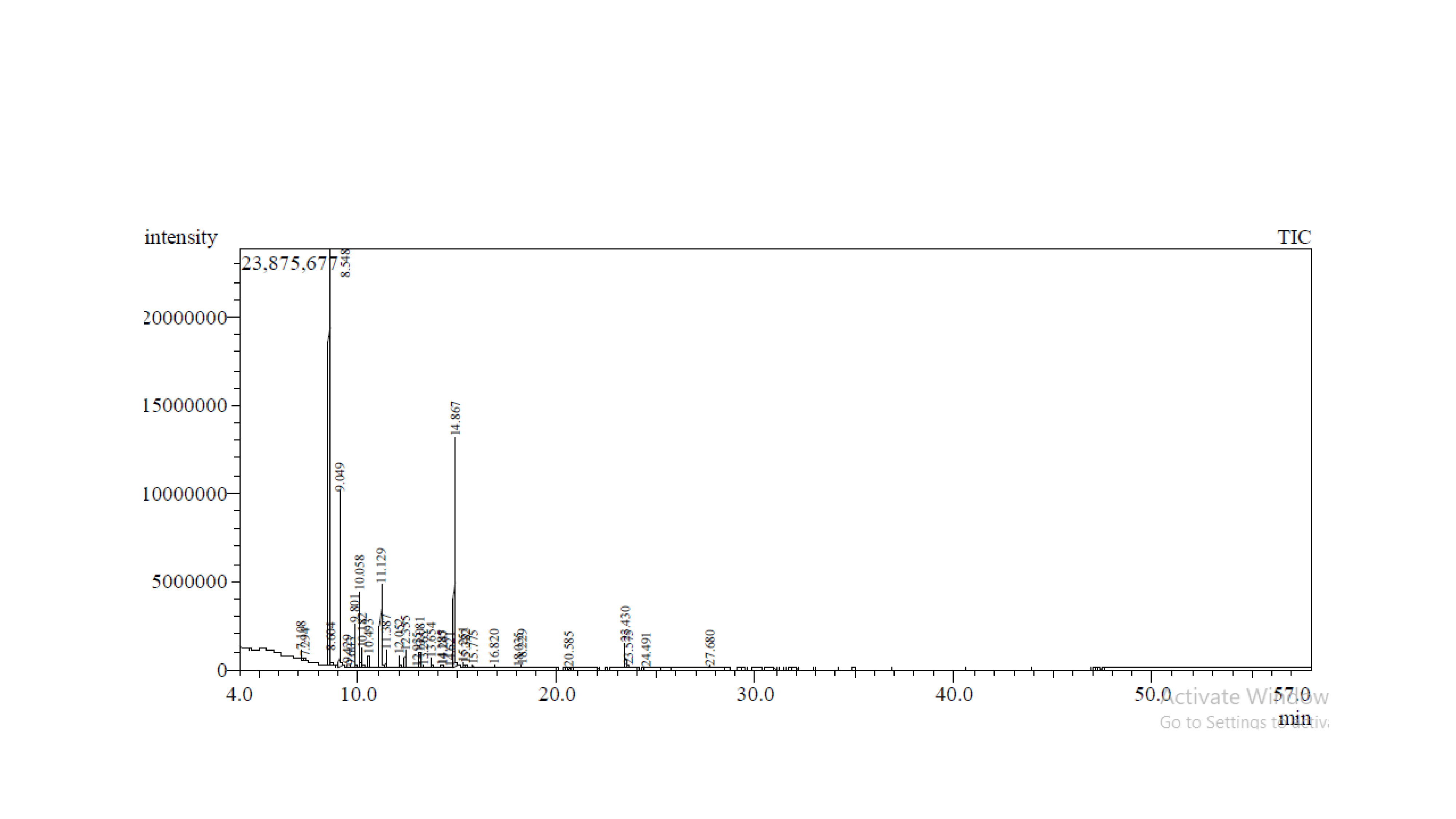

Supplement: Supplementary file 4 [file Image2.TIF]

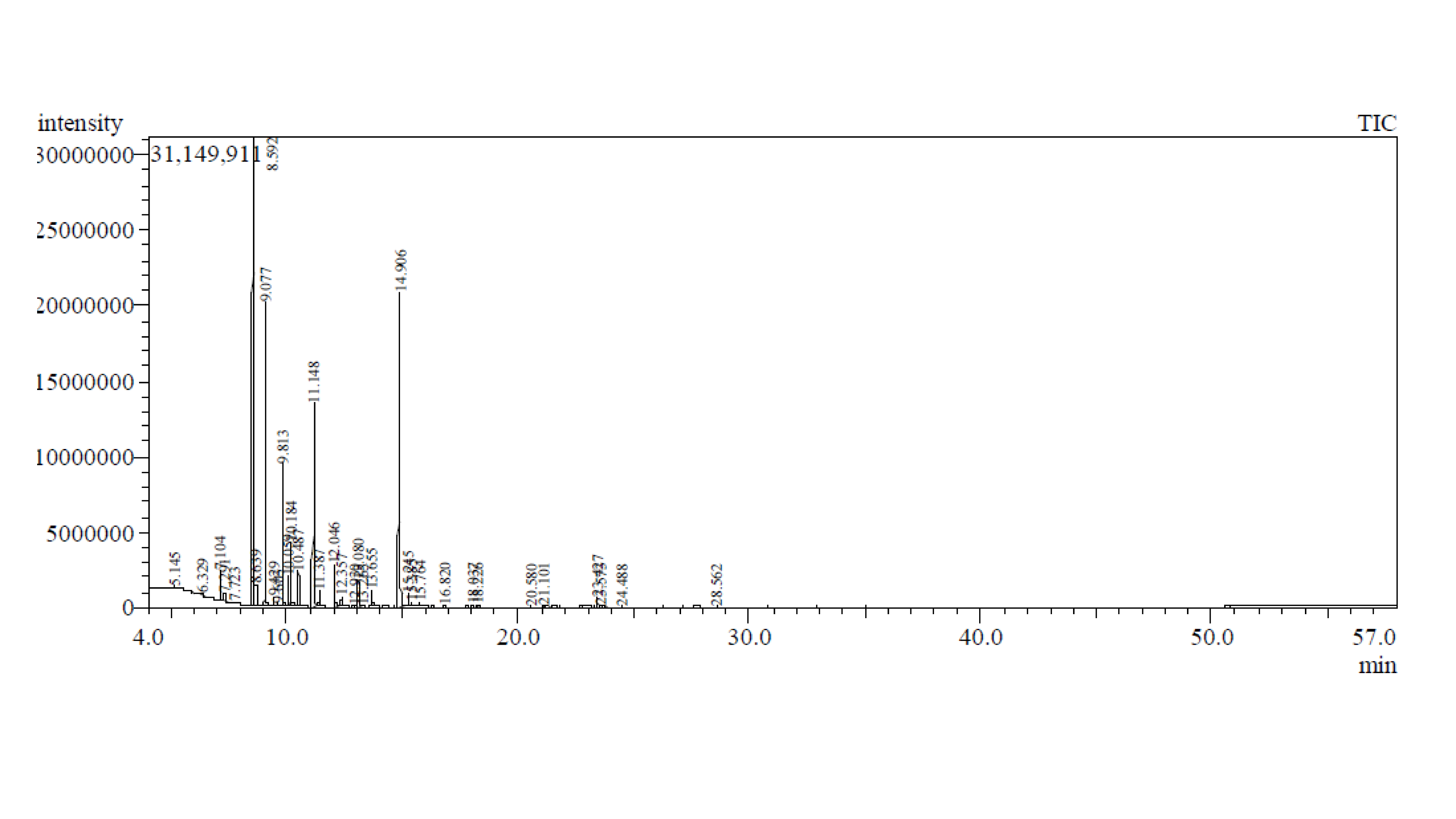

Supplement: Supplementary file 5 [file Image1.TIF]

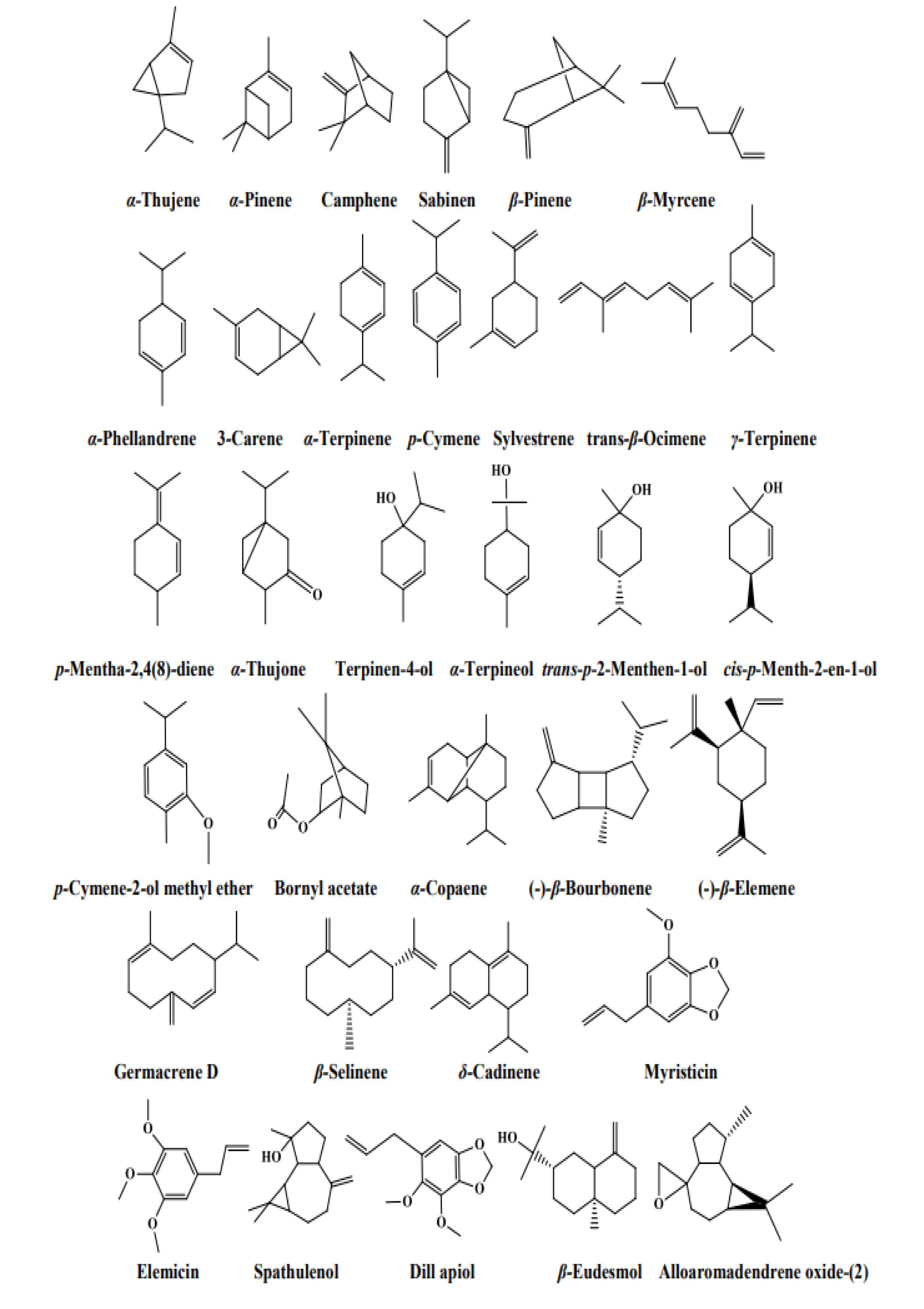

Supplement: Supplementary file 6 [file Image7.TIF]

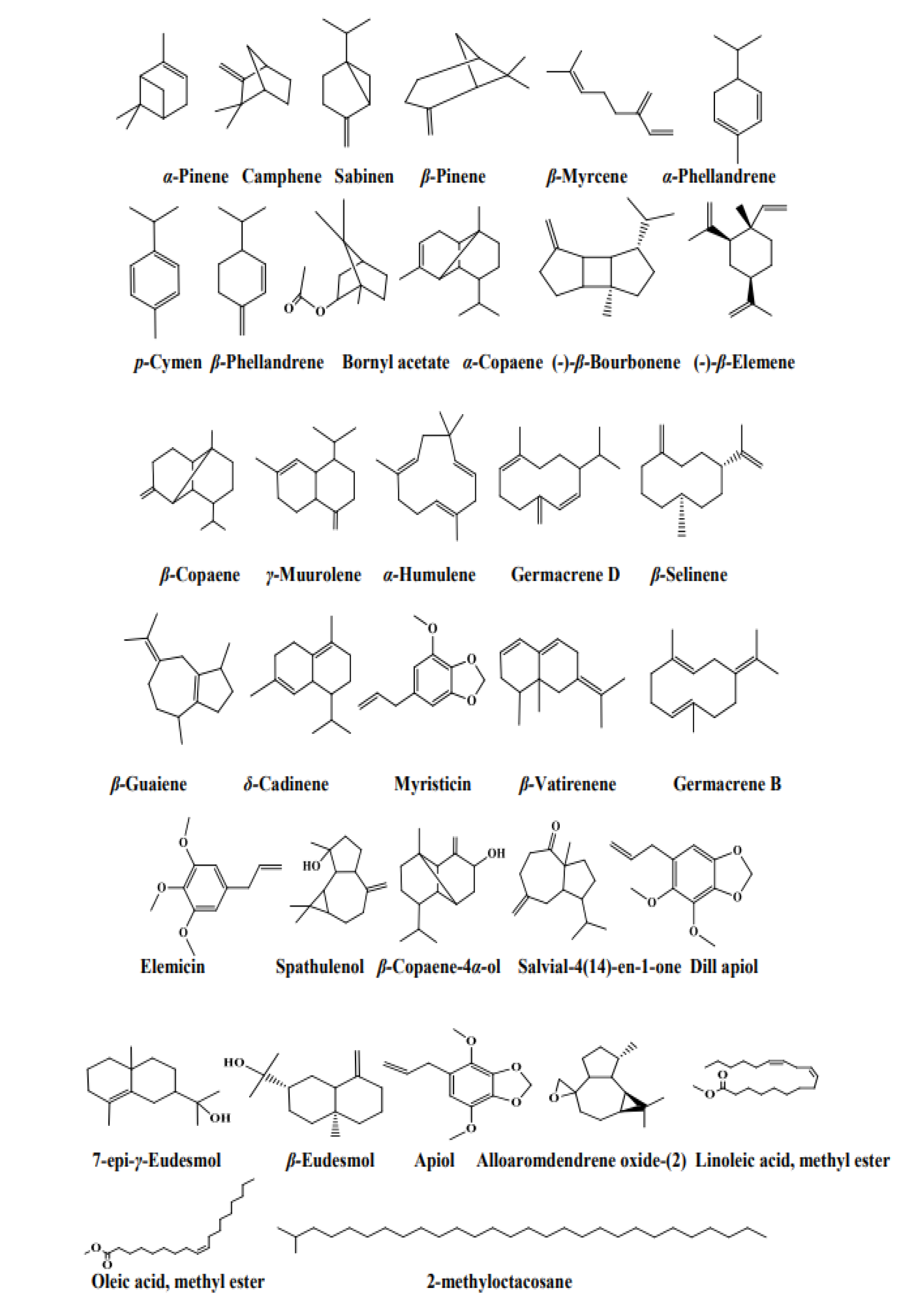

Supplement: Supplementary file 8 [file Image8.TIF]

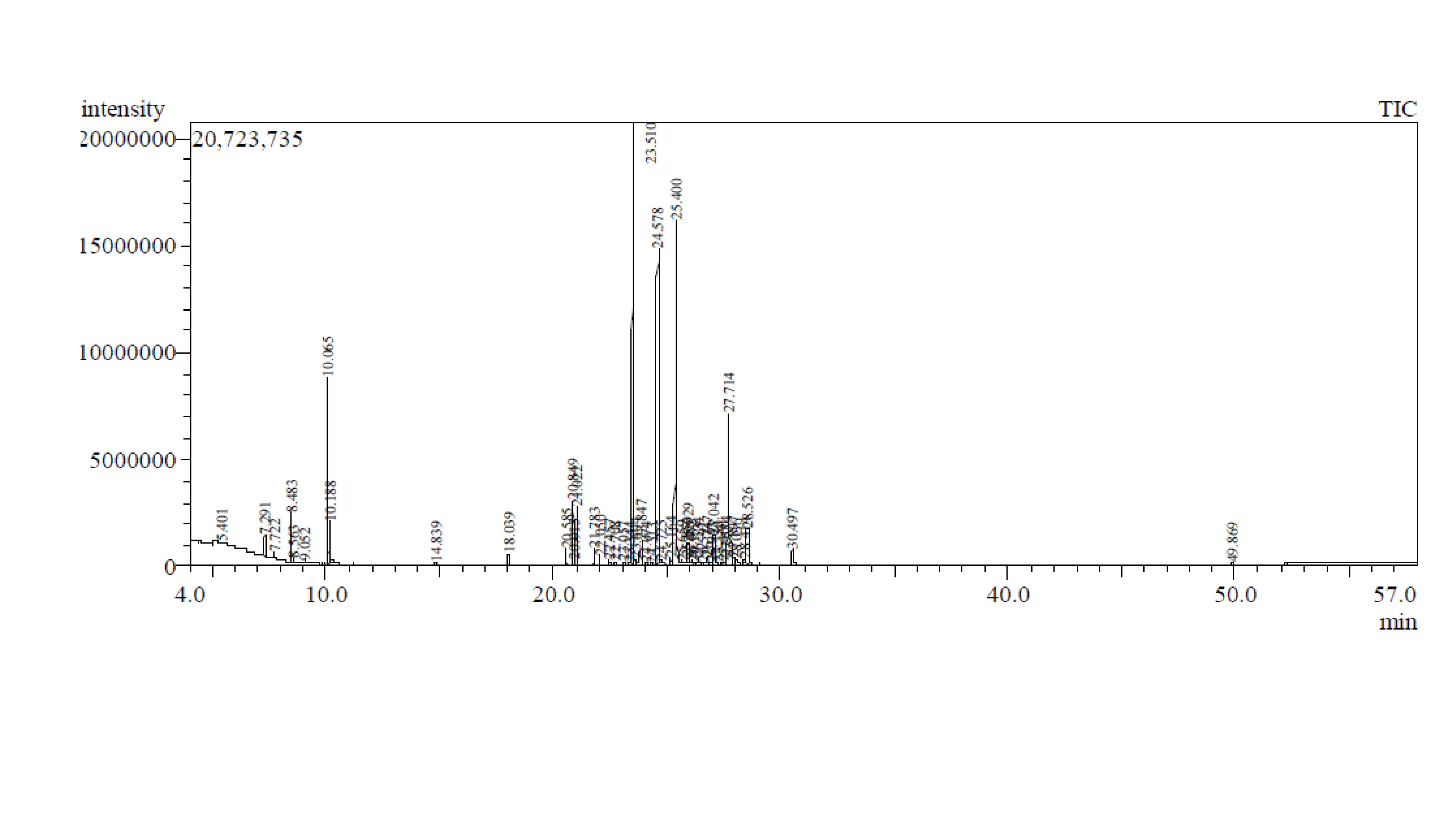

Supplement: Supplementary file 9 [file Image5.TIF]
