# Supplementary material for: Appraisal on the Wound Healing Potential of Deverra tortuosa DC. and Deverra triradiata Hochst Essential Oil Nanoemulsion Topical Preparation
Source: Front Pharmacol. 2022 Jul 26;13:940988. doi: 10.3389/fphar.2022.940988 (PMC9360601; doi:10.3389/fphar.2022.940988)
Supplement: Supplementary file 7 [file DataSheet1.pdf]

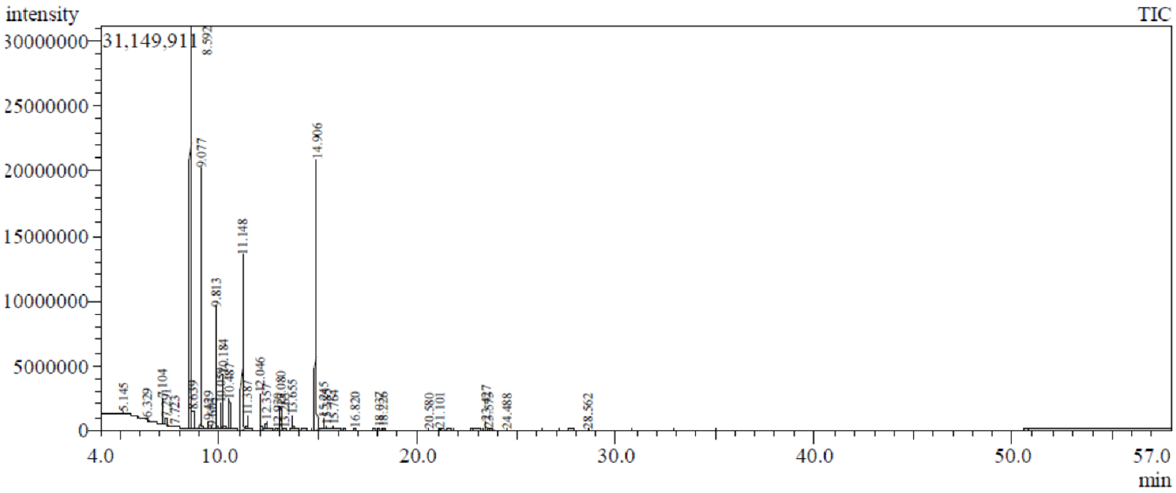

Figure S1: GC/MS Chromatogram for *D. tortuosa* essential oil extracted by HD method

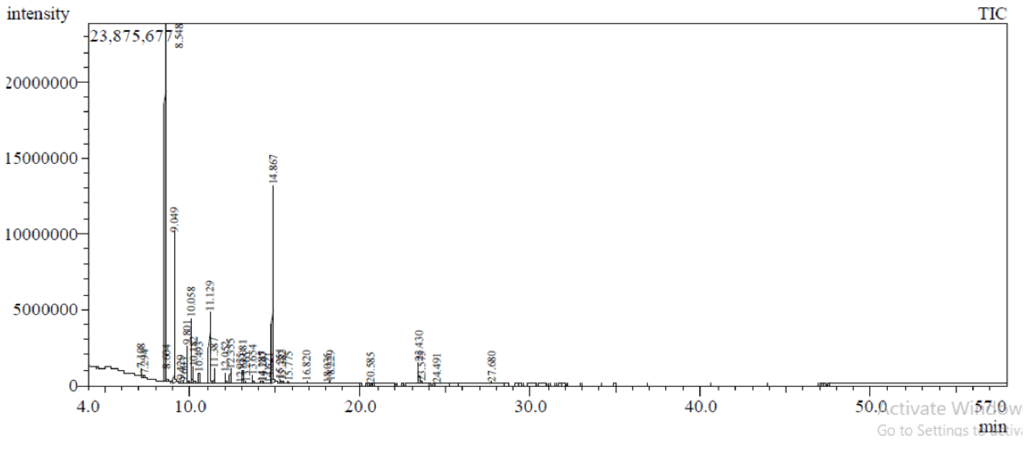

Figure S2: GC/MS Chromatogram for *D. tortuosa* essential oil extracted by MAHD method

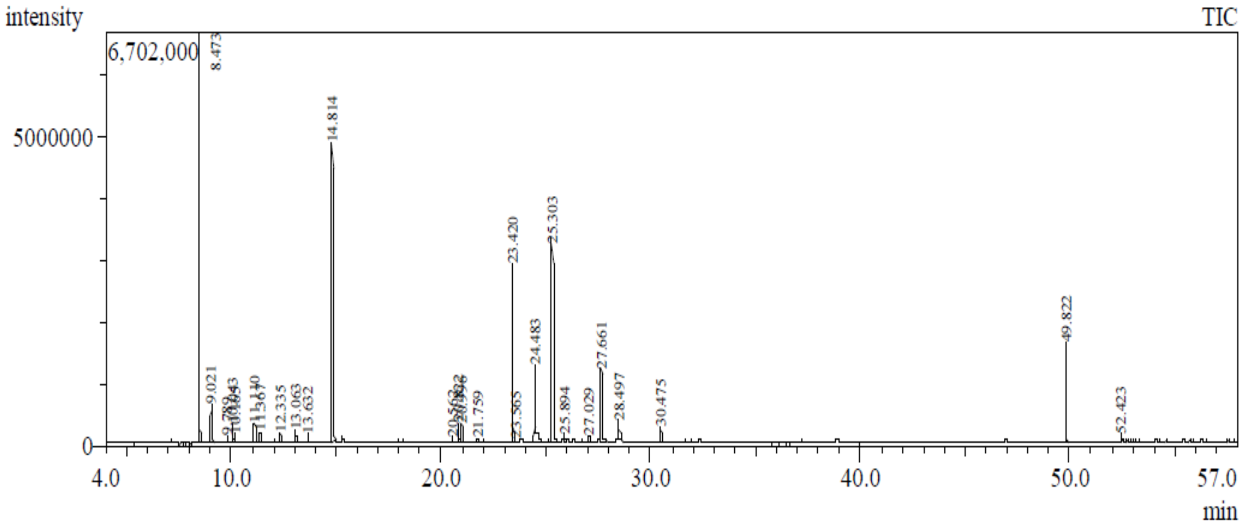

Figure S3: GC/MS Chromatogram for *D. tortuosa* essential oil extracted by SFE method

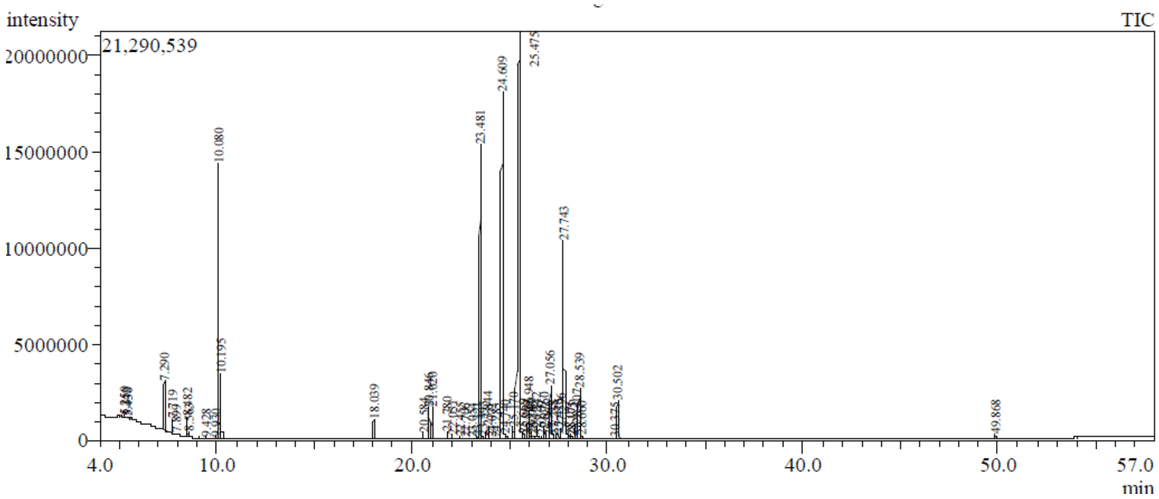

Figure S4: GC/MS Chromatogram for *D. triradiata* extracted by HD method

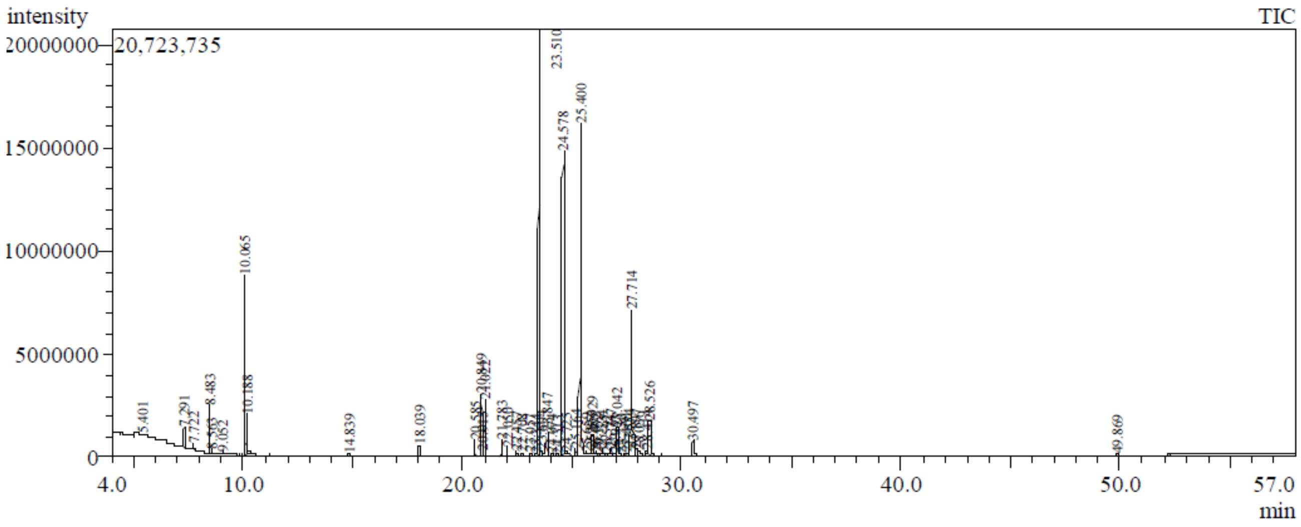

Figure S5: GC/MS Chromatogram for *D. triradiata* extracted by MAHD method

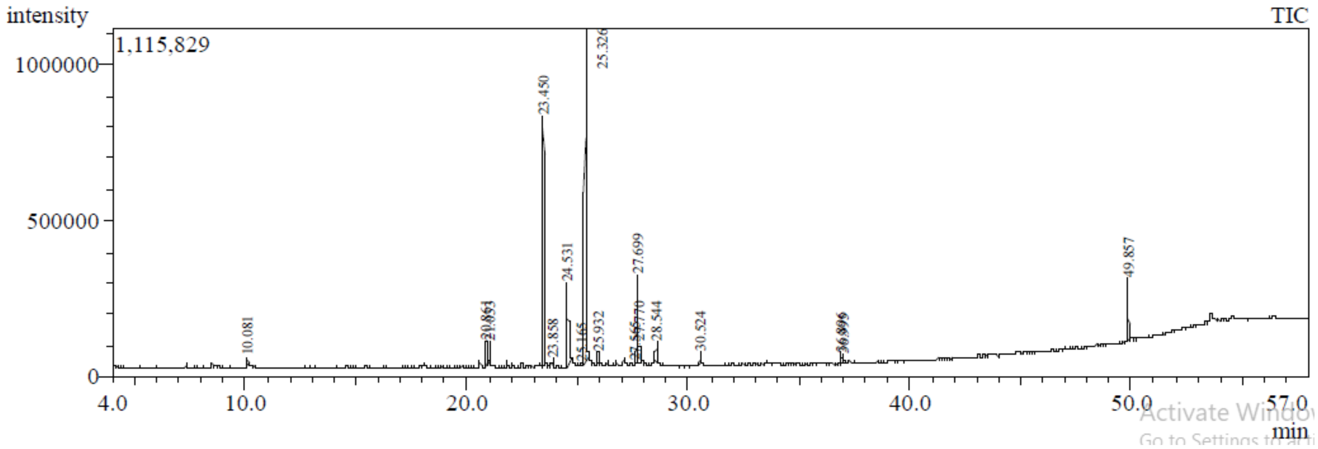

Figure S6: GC/MS Chromatogram for *D. triradiata* extracted by SFE method

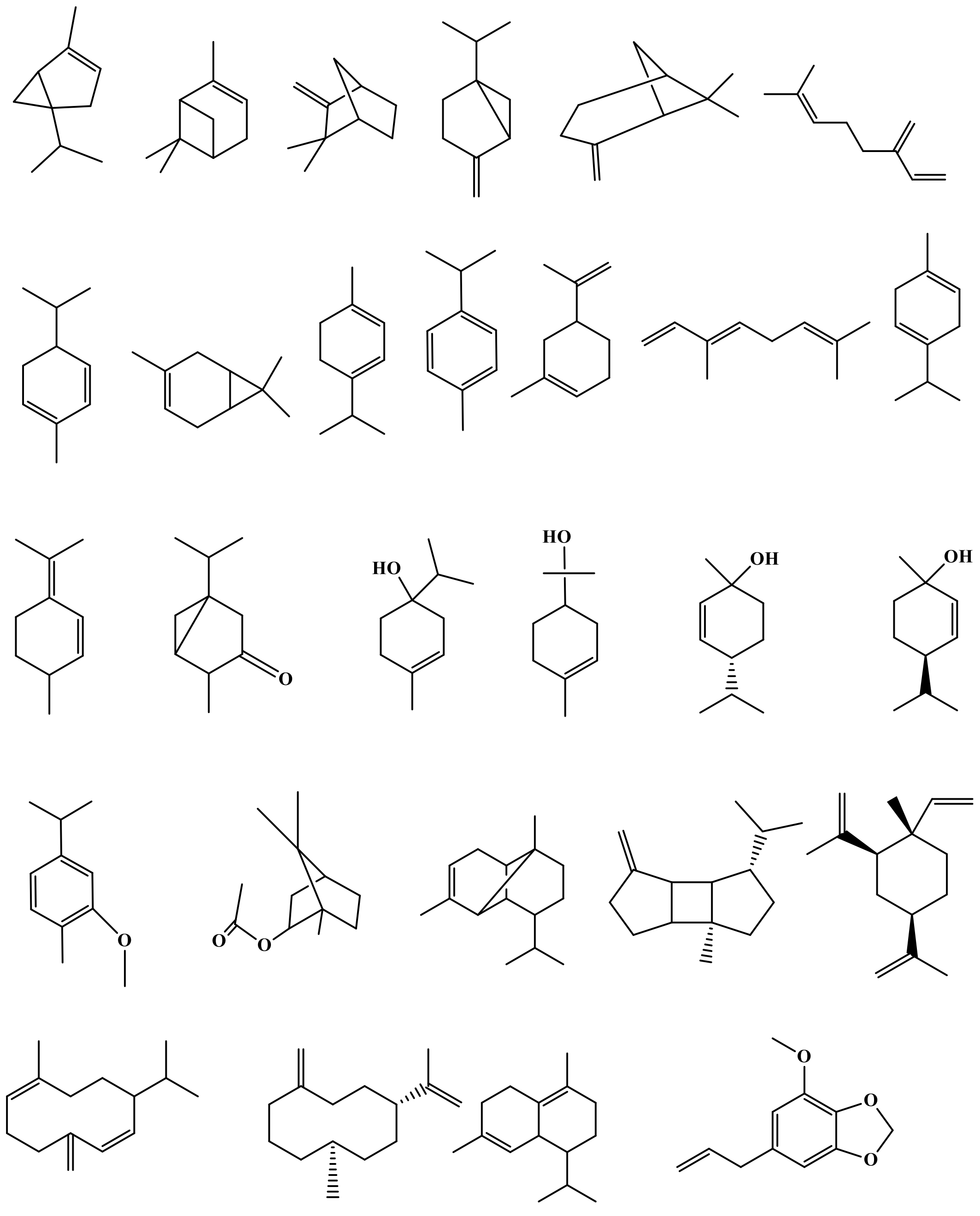

Figure S7: Structures of the compounds detected in the HD, MAHD and SFE oil samples extracted from the aerial parts of *D. tortuosa* DC

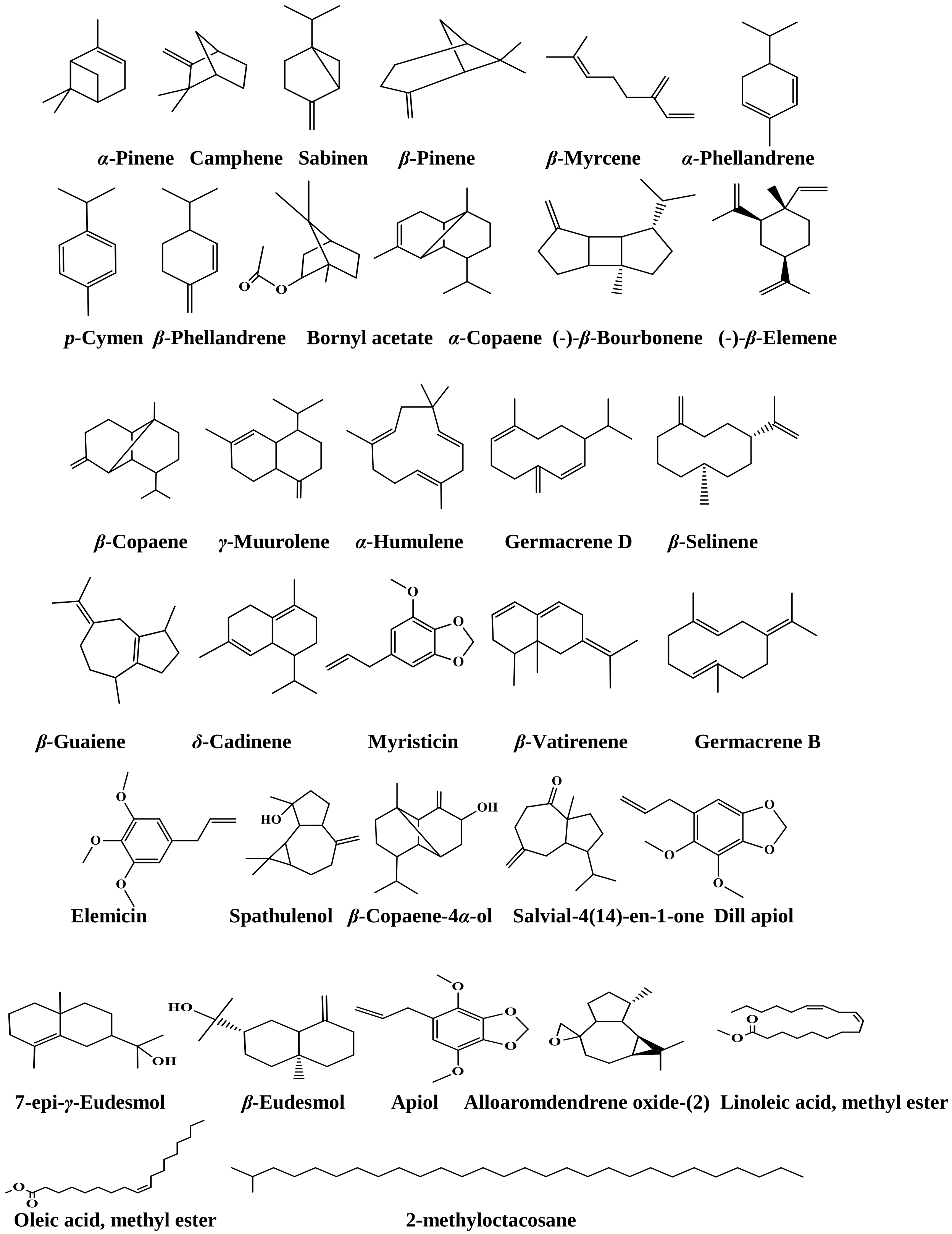

Figure S8: Structures of the compounds detected in the HD, MAHD and SFE oil samples extracted from the aerial parts of *D. triradiata* Hochst.
